# Risk Factors for Lower Extremity Amputation in Patients with End-Stage Kidney Disease: A Nationwide Cohort Study

**DOI:** 10.3390/jcm12175641

**Published:** 2023-08-30

**Authors:** Min Jun Seo, Dong Geon Lee, Se Yun Ko, Ga Yeong Song, Geon Yeong Lee, Sung Hwa Kim, Dae Ryong Kang, Jiye Kim, Jun Young Lee

**Affiliations:** 1 Department of Plastic Surgery, Yonsei University Wonju College of Medicine, Wonju 26426, Republic of Korea; puebjun64@yonsei.ac.kr (M.J.S.); ehdrjs610@yonsei.ac.kr (D.G.L.); 2 Department of Internal Medicine, Yonsei University Wonju College of Medicine, Wonju 26426, Republic of Korea; doffltmnv@naver.com (S.Y.K.); song2491@naver.com (G.Y.S.); kylee4980@yonsei.ac.kr (G.Y.L.); 3 National Health Big Data Clinical Research Institute, Wonju 26426, Republic of Korea; juniver1057@naver.com (S.H.K.); dr.kang@yonsei.ac.kr (D.R.K.); 4 Department of Biostatistics, Yonsei University Wonju College of Medicine, Wonju 26426, Republic of Korea; 5 Department of Precision Medicine, Yonsei University Wonju College of Medicine, Wonju 26426, Republic of Korea; 6 Transplantation Center, Wonju Severance Christian Hospital, Wonju 26426, Republic of Korea; 7 Center of Evidence Based Medicine, Institute of Convergence Science, Yonsei University, Seoul 03722, Republic of Korea

**Keywords:** amputation, end-stage kidney disease, kidney transplantation

## Abstract

Individuals with end-stage kidney disease (ESKD) on dialysis are at a high risk of developing foot ulcerations and undergoing subsequent lower extremity amputation (LEA), which can exert significant impacts on their quality of life and contribute to rising healthcare costs. We aimed to identify risk factors associated with LEA in patients with ESKD to predict LEA progression and eventually prevent it. We used 18 years (2002–2019) of data from the Korean National Health Insurance Service (KNHIS). Data were collected from patients with ESKD who underwent renal replacement therapy (RRT) and had no history of amputation caused by trauma or toxins. The risk factors were compared between patients with or without LEA. We collected data from 220,838 patients newly diagnosed with ESKD, including 6348 in the LEA group and 214,490 in the non-LEA group. The total incidence of LEA was 2.9%. Older age, male gender, lower income, non-metropolitan residence, diabetes mellitus, dialysis treatment (compared to kidney transplantation), microvascular disease, peripheral vascular disease, endovascular procedure, and endovascular operation were associated with an increased risk of LEA. Thus, individuals with ESKD who are at a higher risk for LEA should be closely monitored, and kidney transplantation should be considered as a preventative measure.

## 1. Introduction

With an increase in life expectancy, the global incidence and prevalence of end-stage kidney disease (ESKD) are on the rise [1,2,3]. In the United States, the number of patients newly diagnosed with ESKD increased by 38.2% from 94,466 in 2000 to 130,522 in 2020 [1]. Furthermore, the prevalence of ESKD increased by 107.3%, from 389,592 in 2000 to 807,920 in 2020 [1]. Moreover, the incidence and prevalence of ESKD are particularly high and increasing more rapidly in Asia and Western Pacific countries, including Korea, than in other countries [2,3].

Patients who undergo renal replacement therapy (RRT) have a significantly elevated risk of foot ulceration and lower extremity amputation (LEA). Specifically, the risk for foot ulceration is 7.6 times higher and that for LEA is 15.0 times higher among patients undergoing RRT than for with individuals with stage 3 chronic kidney disease [4]. In 2014, the incidence of LEA was 2.66 per 100 person–years among patients with ESKD in the United States [5]. LEA can lead to reduced quality of life and functional impairment [6], frequent hospitalization [7], higher morbidity and mortality [8], and significant financial burden on the families and the healthcare system [9]. Moreover, higher-level LEA has been related to increased mortality in patients with ESKD compared with that for lower-level LEA [10], emphasizing the importance of addressing the level of LEA comprehensively. LEA prevention can be achieved through early detection, appropriate care, and management of risk factors such as glycemic control and cardiovascular risk factors [11,12]. Therefore, a comprehensive examination of the risk factors associated with LEA in patients with ESKD is crucial.

Previous studies have examined the major risk factors for LEA in patients with ESKD [13,14]. However, there is limited research on the major risk factors for all levels of LEA, such as above-knee (AK), below-knee (BK), and foot/toe (FT) amputation, in such patients with a single criterion and a large sample size over an extended period. Moreover, there are substantial disparities worldwide in the incidence of each RRT modality, including hemodialysis (HD), peritoneal dialysis (PD), and kidney transplantation (KT) [15], as well as the incidence of each level of LEA, including the total (AK, BK, and FT), major (AK and BK), and minor (FT) levels [16]. These disparities highlight the need for detailed investigations into the level of LEA and RRT modalities. Therefore, we aimed to investigate the risk factors for LEA in patients with ESKD and major comorbidities, at all levels of LEA (AK, BK, and FT) and in all modalities of RRT (HD, PD, and KT), by analyzing data from the Korean National Health Insurance Service (KNHIS).

## 2. Materials and Methods

### 2.1. Data Source

We used data from the KNHIS, which covers 97% of the population in South Korea and provides demographic information such as sex, age, residence region, and income quartile, as well as diagnosis and procedure records [17]. Access to KNHIS data is available for academic research in various forms, including the sample research database, customized database, and health disease index database. In this study, we used the customized database provided by KNHIS.

### 2.2. Study Population

**Figure 1:**
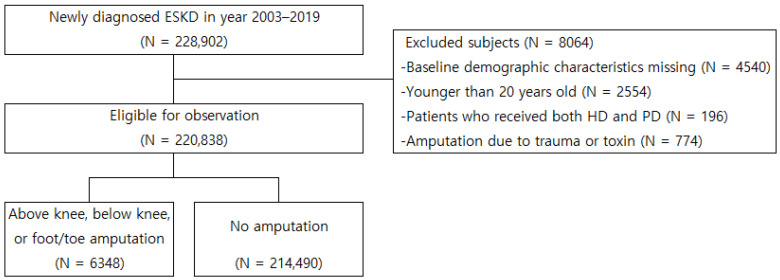
Inclusion and exclusion criteria for observation population. ESKD, end-stage kidney disease; HD, hemodialysis; PD, peritoneal dialysis.

By using specific diagnostic and procedural codes, we extracted information on the patients newly diagnosed with ESKD between 2003 and 2019 (N = 228,902) from the KNHIS database (Figure 1). Patients with missing baseline demographic characteristics (N = 4540), those aged <20 years (N = 2554), and those who underwent dialysis with both HD and PD records (N = 196) or underwent amputation because of trauma or toxins (N = 774) were excluded from the study. The eligible patients were classified into amputation (N = 6348) and no-amputation (N = 214,490) groups (Figure 1).

### 2.3. Covariates and Outcomes

Medical records written within 365 days of the first record of HD, PD, or KT were used to define comorbidities. Variables considered comorbidities are shown in Supplementary Table S1. The Charlson comorbidity index (CCI) was calculated as the weighted sum of 16 categories of comorbidities [18]. The seventh revision of the Korean Standard Classification of Disease code, which is a modification of the 10th revision of the International Classification of Diseases (ICD-10) code, and the procedure code were used to define the variables (Supplementary Table S1). The endpoints of this study were LEA (AK, BK, FT, and combined) and mortality. LEA was further classified into AK, BK, and FT. The procedure codes were used to define AK (N0572), BK (N0573), and FT (N0573 or N0574). Only the first amputation record of the highest-level LEA was considered to avoid a misevaluation of amputation risks.

### 2.4. Statistical Analysis

Baseline characteristics are presented as numbers and percentages for categorical variables and as means and standard deviations (SD) for continuous variables. For comparison, we performed the Fisher’s exact test and Pearson’s chi-square test for categorical variables and the Student’s *t*-test for continuous variables. Multivariate Cox regression analyses were conducted to identify the risk factors for LEA, for each level (AK, BK, and FT) and in total. The results of Cox regression models are presented as hazard ratios (HRs) with 95% confidence intervals (CIs). HRs for each outcome were obtained after being adjusted for baseline characteristics using Cox proportional hazard regression analysis (age, income, residence area, sex, CCI score, past history (diabetes (DM), hypertension, dyslipidemia, chronic pulmonary disease, cancer, major adverse cardiovascular events (MACE), peripheral artery disease (PAD), microvascular disease (MVD), medication (statin, renin-angiotensin-system (RAS) inhibitor, antiplatelet agent, anticoagulant), endovascular procedure history, or endovascular operation history). All statistical analyses were conducted using SAS Enterprise Guide version 7.1 (SAS Inc., Cary, NC, USA). *p*-values < 0.05 were considered statistically significant.

### 2.5. Ethical Statement

This study received ethical approval from the Institutional Review Board of the Wonju Severance Christian Hospital (CR320362). The need for informed consent was waived because the statistical analyses were conducted using KNHIS data, which modifies the information of unidentifiable individuals.

## 3. Results

### 3.1. Incidence and Follow-Up

This study included 220,838 patients who were newly diagnosed with ESKD. Of them, 6348 (2.9%) underwent LEA, with an increasing incidence of lower-level LEA (AK 5.7%, BK 33.3%, and FT 61.0%) (Table 1, Supplementary Table S2). Of these patients, 197,515 (89.4%) and 23,323 (10.6%) were included in the dialysis (HD 81.4% and PD 8.0%) and KT groups, respectively (Supplementary Tables S3 and S4). The dialysis group demonstrated a higher incidence of LEA than the KT group (3.0% vs. 1.7%). Moreover, both groups demonstrated an increasing incidence of lower-level LEA, with a more prominent trend of incidence in the KT group. The incidence of LEA increased from 538.38 (/10^5^ persons) in 2002 to 1163.85 in 2019 (Supplementary Figure S1). The median follow-up time of the patients with ESKD was 7.2 (3.3–12.3) years (median (interquartile range (IQR))). The median time from the commencement of RRT to the occurrence of LEA was 3.6 years, with an IQR of 1.3–6.7 years (AK 4.3 (1.5–6.8), BK 3.9 (1.4–7.1), FT 3.4 (1.2–6.5)).

### 3.2. Baseline Characteristics

Compared with the non-LEA group, the LEA group was younger (58.9 ± 11.1 vs. 61.43 ± 14.94, *p* < 0.0001) and demonstrated a higher percentage of men (70.9% vs. 57.5%, *p* < 0.0001) and a greater proportion of residents of metropolitan areas (69.7% vs. 67.1%, *p* < 0.0001). Moreover, the LEA group exhibited more HD (85.2% vs. 81.3%, *p* < 0.0001) and PD (8.7% vs. 8.0%, *p* = 0.0315) but less KT (6.1% vs. 10.7%, *p* < 0.0001). The proportion of comorbidities, such as coronary artery disease, cerebrovascular disease, diabetes, hypertension, dyslipidemia, microvascular disease, peripheral artery disease, and major adverse cardiovascular events, was higher in the LEA group than the non-LEA group. Compared with the LEA group, non-LEA group received more prescribed antiplatelet agents (5.9% vs. 62.0%, *p* < 0.0001). The LEA group received more endovascular procedures or endovascular surgeries. Although the LEA group had a higher incidence of all causes of death than the non-LEA group (66.9% vs. 47.0%, *p* < 0.0001), the incidence of cardiovascular mortality was not different between two group (8.8% vs. 8.7%, *p* = 0.6641) (Table 1). The event-free survival curve is shown Supplementary Figure S2.

Compared with the KT group, patients in the dialysis group were older in age (63.0 ± 14.3 vs. 47.4 ± 11.4), resided less in metropolitan areas (66.8% vs. 71.1%), were more likely to be the beneficiaries of the National Basic Livelihood (17.2% vs. 12.8%), had higher CCI scores (CCI > 5; 52.6% vs. 40.3%), and generally displayed a trend of higher comorbidity rates (Supplementary Tables S3 and S4). In the KT group, the LEA group was older in age and resided less in metropolitan areas than the non-LEA group. In the dialysis group, the LEA group was younger in age and resided more in metropolitan areas than the non-LEA group. In both the KT and dialysis groups, the LEA group comprised more men (KT 74.8%, dialysis 70.6%) than the non-LEA group (KT 59.3%, dialysis 57.3%). In the KT group, the proportions of members of the fourth income quartile (the wealthiest) and of beneficiaries of National Basic Livelihood (the poorest) were higher in the LEA group than in the non-LEA group. Conversely, the LEA and non-LEA groups did not demonstrate different distributions of income within the dialysis groups.

### 3.3. Risk Factors for LEA

Older age, lower income, non-metropolitan living, male sex, PD, higher comorbidity, DM history, PAD history, MVD history, RAS inhibitor medication prescription, endovascular procedure with history of PAD, and endovascular operation with history of PAD were associated with an increased risk of LEA in ESKD patients. Except PAD history and endovascular procedure history, all of these parameters were also associated with an increased risk of AK, BK, and FT. Older age was associated with an increased risk of high-level LEA (AK). Male sex, DM history, and MVD history were associated with a relatively increased risk of lower-level LEA (FT) (Table 2).

After adjusting for the age, sex, income, residence, RRT modality, and CCI score, medication usage, endovascular procedure history, endovascular surgery history, DM, and MVD were associated with the risk of LEA (AK, BK, FT, and combined) in both KT and dialysis subgroups (Supplementary Table S5). Congestive heart failure, dementia, diabetes with chronic complications, and hemiplegia or paraplegia were associated with an increased risk of LEA in KT patients. Dementia was associated with a relatively increased risk of higher-level amputation (AK, HR 3.91; 95% CI 1.79–8.54), and congestive heart failure (HR 1.30; 95% CI 1.06–1.59), hemiplegia or paraplegia (HR 2.46; 95% CI 1.14–5.32), and diabetes with chronic complications (HR 3.46; 95% CI 2.78–4.32) were associated with a relatively increased risk of lower-level amputation (FT) in KT recipients. In dialysis patients, congestive heart failure, peripheral vascular disease, and diabetes were associated with risk of the LEA. Among dialysis patients, congestive heart failure (HR 1.19; 95% CI 1.15–1.23), diabetes without chronic complications (HR 1.07; 95% CI 1.02–1.12), and diabetes with chronic complications (HR 1.82; 95% CI 1.76–1.88) were associated with lower-level amputation (FT) (Supplementary Table S7).

## 4. Discussion

Through the analysis of a nationwide ESKD cohort database from KNHIS, we identified the risk factors associated with LEA in patients with ESKD. Older age, male sex, lower income, non-metropolitan residence, DM, MVD, peripheral vascular disease (PVD), endovascular procedure, and endovascular operation were associated with the risk of LEA. Specifically, older age and endovascular operation history were associated with a relatively high risk of higher-level LEA, whereas male sex, DM, and MVD were associated with a relatively high risk of lower-level LEA.

In patients with ESKD, KT is more protective against LEA than dialysis, consistent with previous findings (Table 2) [19,20]. Patients receiving dialysis are associated with a higher risk of major LEA [4], consistent with our results. These findings may be attributed to higher serum phosphate levels [21], widespread vascular calcification, and total occlusion of lower extremity arteries in patients receiving dialysis [22,23], resulting in a higher incidence of revascularization and LEA [24,25].

The lower incidence of LEA in the KT group may be influenced by selection bias among KT candidates. Our study indicates a trend of lower comorbidity rates and lower CCI scores in the KT group than in the dialysis groups, which may suggest selection bias (Supplementary Tables S3 and S4). Furthermore, KT recipients may represent a “healthier” population because of potential selection bias [26]. Our data showed that KT recipients who have undergone LEA tend to exhibit a higher prevalence of comorbidities (DM, MVD, coronary artery disease, and PAD) than dialysis patients who have undergone LEA. However, it could be observed that the former group ultimately experiences a lower incidence of high-level amputation. These findings suggest a beneficial effect of KT. Impaired calcium–phosphate metabolism during dialysis is resolved after KT [27]. Therefore, the normalization of serum calcium and phosphate levels after KT may reduce the risk of vascular calcification and occlusion progression, subsequently decreasing the need for revascularization and LEA.

Our study, as well as previous research, has demonstrated that PAD is a risk factor for LEA in patients with ESKD [14]. Moreover, PAD poses a greater risk of higher-level LEA, especially for dialysis patients. The location of arterial involvement in patients with ESKD and PAD may explain these findings. The iliac or femoro-popliteal arteries are affected in 70.1% of patients with ESKD and PAD [22]. Therefore, clinicians should closely monitor the lower extremity arteries, particularly AK, for LEA prevention in patients with dialysis and PAD.

In contrast to our results, higher-level LEA increases the risk of subsequent cardiovascular events such as myocardial infarction (MI) and ischemic stroke [28]. These findings suggest a reciprocal relationship between MACE and higher-level LEA, indicating the possibility of “systemic macrovascular damage” in patients with ESKD that can lead to both conditions simultaneously. Furthermore, subclinical atherosclerosis at the carotid, femoral, and coronary arteries is common in the general population [29], and patients with ESKD have a higher prevalence of PAD than those without ESKD [30]. Moreover, PAD can indicate similarly sized vascular diseases, such as MI and ischemic stroke [29,31]. Therefore, clinicians should consider “systemic macrovascular damage” in patients with ESKD and MI or ischemic stroke because it may increase the risk of higher-level LEA and vice versa. However, further research is necessary to understand the causal relationship and the mechanisms underlying “systemic macrovascular damage”.

Our study and previous research have consistently identified DM as a significant risk factor for LEA in patients with ESKD, with a considerably higher risk than other comorbidities [14]. Furthermore, DM with complications poses a significantly greater risk of LEA than that without complications. The reason for these findings may be attributed to the synergistic effects of DM-related complications such as circulatory, neurologic, and other conditions. Moreover, the coexistence of MVD and PAD in patients with DM synergistically increases the risk of LEA [32]. In addition, DM and MVD are risk factors for LEA, particularly BK and FT. This finding may be attributed to the sensory damage caused by DM and MVD, particularly below the knee, resulting in ulcers, deformities, and other complications [33,34]. Therefore, clinicians should closely monitor the lower extremities, particularly BK and FT, to prevent LEA in patients with ESKD along with DM or MVD.

Similar to other studies, our study showed that dyslipidemia was associated with a lower risk of LEA in patients with ESKD [31]. In some studies, statin has been reported to be associated with a lower rate of amputation; however, like in our study, those studies could not know the statin dose or exact duration of prescription [28,35,36]. More research is needed to evaluate the relationship between amputation and statin. Furthermore, our study and previous research have consistently found that hypertension is not a significant risk factor for LEA in ESKD patients [14,37]. Therefore, dyslipidemia and hypertension do not appear to be significant risk factors for LEA.

Our study and previous studies have identified middle age and male sex as the risk factors for LEA in patients with ESKD [5,38]. Interestingly, such patients with chronic pulmonary disease, cancer, and old age (>60 years) have a decreased risk of LEA. These findings may be attributed to older adults prioritizing the management of chronic diseases over receiving LEA in ESKD.

Patients with ESKD who are beneficiaries of National Basic Livelihood have an increased risk of LEA. Patients with HD on Medicaid or who are privately uninsured have a higher risk of LEA than those on Medicare or who are privately insured [39]. These findings may be attributed to the inability of poor individuals to receive adequate healthcare management early, which can lead to delayed treatment and eventually result in conditions severe enough to require LEA. Furthermore, poor individuals are unable to receive adequate healthcare and proper treatment [40] and have a higher risk of LEA [41].

Previous studies have provided inconsistent evidence regarding socioeconomic status and metropolitan residency as risk factors for LEA [41,42,43,44]. Whereas some studies suggest that socioeconomic status is a significant risk factor for LEA [41,42], others indicate that urban residence plays an important role [43,44]. Our study was conducted in South Korea and suggests that relatively poor patients with ESKD (income quartiles 1–3) and those residing in metropolitan areas have a negligible risk of LEA. Thus, in patients with ESKD, socioeconomic status (except in beneficiaries of National Basic Livelihood) and metropolitan residency may not be significant risk factors for LEA in South Korea, and this relationship may vary across countries.

### 4.1. Limitations

Our study has a few limitations. First, crucial physiological variables, including glomerular filtration rate, blood pressure, low-density lipoprotein, high-density lipoprotein, triglycerides, blood glucose, and glycated hemoglobin are unavailable in the KNHIS database and thus are not included in the analysis. Second, the use of ICD-10 codes to define comorbidities and calculate the CCI score may have resulted in a misclassification bias. This is because these codes may not accurately represent the underlying disease. Third, the sole reliance on ICD-10 codes and procedure codes to define ESKD and comorbidities without considering the severity, or, duration may limit accuracy when identifying the impact of these conditions on the risk of LEA. Finally, the failure to consider foot ulceration, deformities, and prior LEA history, which are important risk factors for LEA [13,14], may underestimate their impact on the risk of LEA.

### 4.2. Strengths

In this novel nationwide cohort study, we examined the long-term risks of LEA in patients with ESKD over a 17-year period, analyzing them by the modality of RRT, level of LEA, and major comorbidities. Throughout this study, we found that older age and endovascular surgical treatment were associated with higher-level amputation. Male gender, DM, and MVD history were associated with lower-level amputation. The substantial sample size, comprising nearly all patients with ESKD in South Korea, adds significant value to the study.

## 5. Conclusions

In this nationwide cohort study, we identified various risk factors associated with LEA, including dialysis, PAD, DM, MVD, age, sex, income, and residency, in patients with ESKD. Such patients with older age, demonstrated a higher risk of higher-level LEA, whereas those with DM and MVD demonstrated a higher risk of lower-level LEA. Therefore, patients with ESKD along with older ageshould be cautious about higher-level LEA, whereas those with DM or MVD should be cautious about lower-level LEA. Furthermore, our findings recommend kidney transplantation as a preventative measure against LEA and emphasizes the importance of regular monitoring, not only for LEA but also for other diseases, such as PVD and congestive heart failure.

## Figures and Tables

**Table 1:** Patient characteristics.

		Control	LEA (Total)		LEA (Total)			
		(N = 214,490)	(N = 6348)	AK (N = 363)	BK (N = 2113)	FT (N = 3872)	*p*-Value *	*p*-Value **
		N (%)	N (%)	N (%)	N (%)	N (%)		
Age (mean, SD)		61.43 (14.94)	58.88 (11.09)	58.81 (10.55)	58.07 (10.58)	59.33 (11.39)	<0.0001	<0.0001
Age								
	20–29	5116 (2.4)	27 (0.4)	1 (0.3)	13 (0.6)	13 (0.3)	<0.0001	<0.0001
	30–39	13,620 (6.3)	213 (3.4)	11 (3.0)	67 (3.2)	135 (3.5)		
	40–49	28,822 (13.4)	1079 (17.0)	63 (17.4)	365 (17.3)	651 (16.8)		
	50–59	44,360 (20.7)	1973 (31.1)	115 (31.7)	701 (33.2)	1157 (29.9)		
	60–69	50,089 (23.4)	1904 (30.0)	111 (30.6)	679 (32.1)	1114 (28.8)		
	>70	72,483 (33.8)	1152 (18.1)	62 (17.1)	288 (13.6)	802 (20.7)		
Sex								
	Male	123,335 (57.5)	4499 (70.9)	241 (66.4)	1515 (71.7)	2743 (70.8)	<0.0001	0.1205
	Female	91,155 (42.5)	1849 (29.1)	122 (33.6)	598 (28.3)	1129 (29.2)		
Residence								
	Metropolitan	144,003 (67.1)	4424 (69.7)	245 (67.5)	1476 (69.9)	2703 (69.8)	<0.0001	0.6434
	Non-metropolitan	70,487 (32.9)	1924 (30.3)	118 (32.5)	637 (30.1)	1169 (30.2)		
Income								
	0 ^†^	35,680 (16.6)	1185 (18.7)	85 (23.4)	429 (20.3)	671 (17.3)	<0.0001	0.0046
	1st	35,160 (16.4)	1023 (16.1)	66 (18.2)	340 (16.1)	617 (15.9)		
	2nd	34,949 (16.3)	1051 (16.6)	63 (17.4)	342 (16.2)	646 (16.7)		
	3rd	44,240 (20.6)	1323 (20.8)	73 (20.1)	439 (20.8)	811 (20.9)		
	4th	64,461 (30.1)	1766 (27.8)	76 (20.9)	563 (26.6)	1127 (29.1)		
ESKD (RRT)								
	HD	174,427 (81.3)	5409 (85.2)	316 (87.1)	1825 (86.4)	3268 (84.4)	<0.0001	0.0726
	PD	17,125 (8.0)	554 (8.7)	38 (10.5)	213 (10.1)	303 (7.8)	0.0315	0.0061
	KT	22,938 (10.7)	385 (6.1)	9 (2.5)	75 (3.5)	301 (7.8)	<0.0001	<0.0001
Past history								
	CAD	63,419 (29.6)	2430 (38.3)	136 (37.5)	788 (37.3)	1506 (38.9)	<0.0001	0.4511
	CVD	44,785 (20.9)	1505 (23.7)	113 (31.1)	496 (23.5)	896 (23.1)	<0.0001	0.0027
	DM	135,546 (63.2)	5851 (92.2)	311 (85.7)	1960 (92.8)	3580 (92.5)	<0.0001	<0.0001
	Hypertension	178,956 (83.4)	5637 (88.8)	317 (87.3)	1860 (88.0)	3460 (89.4)	<0.0001	0.1939
	Dyslipidemia	127,335 (59.4)	4069 (64.1)	221 (60.9)	1324 (62.7)	2524 (65.2)	<0.0001	0.0632
	CCI score (mean, SD)	4.85 (2.06)	5.03 (1.81)	5.25 (1.98)	4.98 (1.84)	5.04 (1.77)	<0.0001	<0.0001
	CCI ≥ 5	109,679 (51.1)	3639 (57.3)	223 (61.4)	1173 (55.5)	2243 (57.9)	<0.0001	0.052
	MVD	81,573 (38.0)	4321 (68.1)	225 (62.0)	1462 (69.2)	2634 (68.0)	<0.0001	0.0246
	PND	32,264 (15.0)	2049 (32.3)	114 (31.4)	710 (33.6)	1225 (31.6)	<0.0001	0.2799
	Retinopathy	68,126 (31.8)	3670 (57.8)	178 (49.0)	1253 (59.3)	2239 (57.8)	<0.0001	0.0012
	CPD	62,538 (29.2)	1654 (26.1)	89 (24.5)	526 (24.9)	1039 (26.8)	<0.0001	0.2077
	Cancer	22,854 (10.7)	303 (4.8)	14 (3.9)	100 (4.7)	189 (4.9)	<0.0001	0.6778
	Depression	21,670 (10.1)	615 (9.7)	32 (8.8)	198 (9.4)	385 (9.9)	0.2794	0.6546
	PAD	44,204 (20.6)	1907 (30.0)	124 (34.2)	597 (28.3)	1186 (30.6)	<0.0001	0.0337
	PVD	40,936 (19.1)	1771 (27.9)	116 (32.0)	553 (26.2)	1102 (28.5)	<0.0001	0.0349
	LER	5309 (2.5)	356 (5.6)	23 (6.3)	108 (5.1)	225 (5.8)	<0.0001	0.4383
	MACE	41,962 (19.6)	1615 (25.4)	111 (30.6)	573 (27.1)	931 (24.0)	<0.0001	0.0023
	PCI	7031 (3.3)	387 (6.1)	24 (6.6)	133 (6.3)	230 (5.9)	<0.0001	0.7873
	CABG	1137 (0.5)	90 (1.4)	7 (1.9)	42 (2.0)	41 (1.1)	<0.0001	0.0103
	MI	11,157 (5.2)	468 (7.4)	28 (7.7)	170 (8.0)	270 (7.0)	<0.0001	0.3062
	Ischemic stroke	27,141 (12.7)	998 (15.7)	73 (20.1)	354 (16.8)	571 (14.7)	<0.0001	0.0076
	Hemorrhagic stroke	4092 (1.9)	73 (1.1)	9 (2.5)	19 (0.9)	45 (1.2)	<0.0001	0.0331
	Medication							
	RAS inhibitor	150,736 (70.3)	5113 (80.5)	289 (79.6)	1693 (80.1)	3131 (80.9)	<0.0001	0.7082
	Antiplatelet	12,567 (5.9)	3938 (62.0)	230 (63.4)	1260 (59.6)	2448 (63.2)	<0.0001	0.0205
	Anticoagulant	7225 (3.4)	227 (3.6)	14 (3.9)	71 (3.4)	142 (3.7)	0.367	0.7937
	Surgery							
	Endovascular procedure	54,099 (25.2)	3087 (48.6)	181 (49.9)	1096 (51.9)	1810 (46.7)	<0.0001	0.0007
	Endovascular operation	204 (0.1)	98 (1.5)	18 (5.0)	44 (2.1)	36 (0.9)	<0.0001	<0.0001
	All causes of death	100,729 (47.0)	4245 (66.9)	303 (83.5)	1616 (76.5)	2326 (60.1)	<0.0001	<0.0001
	CV mortality	18,886 (8.8)	549 (8.7)	41 (11.3)	190 (9.0)	318 (8.2)	0.6641	0.2051

* Comparison between non-amputation and amputation groups. ** Comparison between AK, BK, and FT groups. ^†^ Beneficiaries of National Basic Livelihood. AK, above-knee amputation; BK, below-knee amputation; CABG, coronary artery bypass graft; CAD, coronary artery disease; CCI, Charlson’s comorbidity index; CPD, chronic pulmonary disease; CVD, cerebrovascular disease; DM, diabetes mellitus; ESKD, end-stage kidney disease; FT, foot or toe amputation; HD, hemodialysis; KT, kidney transplantation; LEA, lower extremity amputation; LER, lower extremity revascularization; MACE, major adverse cardiovascular events; MI, myocardial infarction; MVD, microvascular disease; PAD, peripheral artery disease; PCI, percutaneous coronary intervention; PD, peritoneal dialysis; PND, peripheral nerve disease; PVD, peripheral vascular disease; RAS; renin–angiotensin system, RRT, renal replacement therapy; SD, standard deviation.

**Table 2:** Risk factors for lower extremity amputation.

		Multivariable Cox Regression (Adjusted)			
		LEA (Total)	AK	BK	FT
	Variables	HR (95% CI)	HR (95% CI)	HR (95% CI)	HR (95% CI)
Age (mean, SD)					
	20–29	0.15 (0.13–0.18)	0.15 (0.13–0.18)	0.16 (0.13–0.19)	0.15 (0.12–0.18)
	30–39	0.33 (0.30–0.35)	0.32 (0.29–0.34)	0.32 (0.29–0.34)	0.32 (0.30–0.35)
	40–49	0.68 (0.65–0.71)	0.65 (0.62–0.68)	0.66 (0.63–0.69)	0.67 (0.64–0.69)
	50–59	1	1	1	1
	60–69	1.39 (1.34–1.43)	1.46 (1.41–1.51)	1.43 (1.38–1.48)	1.41 (1.37–1.46)
	>70	1.63 (1.58–1.69)	1.71 (1.64–1.77)	1.65 (1.60–1.71)	1.68 (1.63–1.74)
Income	0 ^†^	1.46 (1.41–1.51)	1.43 (1.38–1.48)	1.45 (1.39–1.50)	1.43 (1.38–1.48)
	1	1.02 (0.98–1.06)	1.00 (0.96–1.05)	1.01 (0.97–1.05)	1.01 (0.97–1.05)
	2	1.03 (1.00–1.07)	1.04 (1.00–1.08)	1.03 (0.99–1.07)	1.03 (0.99–1.07)
	3	1.03 (0.99–1.06)	1.03 (0.99–1.07)	1.03 (0.99–1.07)	1.03 (0.99–1.06)
	4	1	1	1	1
Residence	Metropolitan	0.93 (0.90–0.95)	0.91 (0.88–0.93)	0.91 (0.89–0.94)	0.92 (0.90–0.94)
	Non-metropolitan	1	1	1	1
Sex	Male	1.23 (1.20–1.26)	1.15 (1.12–1.18)	1.18 (1.15–1.21)	1.20 (1.17–1.23)
	Female	1	1	1	1
ESKD (RRT)	HD	1	1	1	1
	PD	1.35 (1.29–1.40)	1.34 (1.28–1.40)	1.35 (1.30–1.41)	1.33 (1.28–1.39)
	KT	0.22 (0.21–0.24)	0.13 (0.12–0.15)	0.15 (0.13–0.46)	0.21 (0.20–0.23)
CCI score	≥5	1.06 (1.03–1.09)	1.06 (1.02–1.09)	1.05 (1.02–1.09)	1.06 (1.03–1.09)
	<5	1	1	1	1
Past history	DM	1.65 (1.60–1.70)	1.42 (1.38–1.47)	1.51 (1.46–1.56)	1.57 (1.52–1.62)
	Hypertension	1.01 (0.97–1.04)	1.01 (0.97–1.05)	1.01 (0.97–1.05)	1.01 (0.64–1.05)
	Dyslipidemia	0.78 (0.76–0.80)	0.73 (0.71–0.75)	0.75 (0.73–0.77)	0.77 (0.75–0.79)
	CPD	0.96 (0.93–0.98)	0.95 (0.92–0.98)	0.95 (0.92–0.98)	0.96 (0.93–0.98)
	Cancer	0.79 (0.76–0.83)	0.81 (0.77–0.85)	0.81 (0.77–0.85)	0.80 (0.76–0.84)
	MACE	0.33 (0.31–0.34)	0.14 (0.13–0.15)	0.21 (0.20–0.22)	0.25 (0.24–0.26)
	PAD	1.04 (1.01–1.07)	0.95 (0.92–0.98)	0.97 (0.94–1.01)	1.01 (0.98–1.04)
	MVD	1.37 (1.34–1.41)	1.30 (1.20–1.37)	1.29 (1.25–1.32)	1.33 (1.29–1.36)
Medication	Statin	0.81 (0.75–0.87)	0.74 (0.68–0.81)	0.77 (0.70–0.83)	0.79 (0.73–0.86)
	RAS inhibitor	1.05 (1.02–1.08)	1.04 (1.00–1.07)	1.05 (1.01–1.08)	1.05 (1.01–1.08)
	Antiplatelet	0.97 (0.94–0.99)	0.91 (0.88–0.94)	0.92 (0.90–0.95)	0.95 (0.92–0.98)
	Anticoagulant	1.08 (1.00–1.17)	0.94 (0.85–1.03)	0.98 (0.90–1.07)	1.04 (0.96–1.13)
Surgery or procedure	Endovascular procedure	1.06 (1.03–1.09)	1.01 (0.98–1.04)	1.04 (1.01–1.06)	1.04 (1.01–1.06)
	Bypass operation	2.06 (1.75–2.43)	1.83 (1.43–2.34)	1.97 (1.60–2.43)	1.75 (1.41–2.18)

^†^ Beneficiaries of National Basic Livelihood. AK, above-knee amputation; BK, below-knee amputation; CCI, Charlson’s comorbidity index; CI, confidence interval; CPD, chronic pulmonary disease; DM, diabetes mellitus; ESKD, end-stage kidney disease; FT, foot or toe amputation; HD, hemodialysis; HR, hazard ratio; KT, kidney transplantation; LEA, lower extremity amputation; MACE, major adverse cardiovascular events; MVD, microvascular disease; PAD, peripheral artery disease; PD, peritoneal dialysis; RAS, Renin-Angiotensin System; RRT, renal replacement therapy.

## Data Availability

KNHIS provides data with the approval of KNHIS through the Korean National Health Insurance Sharing Service (http://nhiss.nhis.or.kr, accessed on 23 August 2023).

## Supplementary Materials

The following supporting information can be downloaded at: https://www.mdpi.com/article/10.3390/jcm12175641/s1, Figure S1. Incidence of Lower Extremity Amputation; Table S1. Definition of Variables; Table S2. Charlson’s Comorbidity Index; Table S3. Patient Characteristics (Subgroup); Table S4. Charlson’s Comorbidity Index (Subgroup); Table S5. Multivariable Cox Regression (Subgroup); Table S6. Multivariable Cox Regression (CCI); Table S7. Multivariable Cox Regression (Subgroup); Figure S2. Event free survival curve of the entire group and subgroups.

## References

[1] USRDS 2022 USRDS Annual Report, End Stage Renal Disease, Chapter 1. Incidence, Prevalence, Patient Characteristics, and Treatment Modalities. https://usrds-adr.niddk.nih.gov/2022/end-stage-renal-disease/1-incidence-prevalence-patient-characteristics-and-treatment-modalities.

[2] Prasad N., Jha V. (2015). Hemodialysis in asia. Kidney Dis..

[3] Cheng H.-T., Xu X., Lim P.S., Hung K.-Y. (2021). Worldwide epidemiology of diabetes-related end-stage renal disease, 2000–2015. Diabetes Care.

[4] Otte J., van Netten J.J., Woittiez A.-J.J. (2015). The association of chronic kidney disease and dialysis treatment with foot ulceration and major amputation. J. Vasc. Surg..

[5] Franz D., Zheng Y., Leeper N.J., Chandra V., Montez-Rath M., Chang T.I. (2018). Trends in rates of lower extremity amputation among patients with end-stage renal disease who receive dialysis. JAMA Intern. Med..

[6] Coffey L., Gallagher P., Horgan O., Desmond D., MacLachlan M. (2009). Psychosocial adjustment to diabetes—Related lower limb amputation. Diabet. Med..

[7] Lavery L.A., Lavery D.C., Hunt N.A., La Fontaine J., Ndip A., Boulton A.J. (2015). Amputations and foot-related hospitalisations disproportionately affect dialysis patients. Int. Wound J..

[8] Hickson L.J., Rule A.D., Thorsteinsdottir B., Shields R.C., Porter I.E., Fleming M.D., Ubl D.S., Crowson C.S., Hanson K.T., Elhassan B.T. (2018). Predictors of early mortality and readmissions among dialysis patients undergoing lower extremity amputation. J. Vasc. Surg..

[9] Takemoto Y., Naganuma T., Nakanishi T., Kuragano T. (2019). Economic issues of chronic kidney disease and end-stage renal disease. CKD-Associated Complications: Progress in the Last Half Century.

[10] Lavery L.A., Hunt N.A., Ndip A., Lavery D.C., Van Houtum W., Boulton A.J.M. (2010). Impact of chronic kidney disease on survival after amputation in individuals with diabetes. Diabetes Care.

[11] Jeffcoate W.J., Van Houtum W.H. (2004). Amputation as a marker of the quality of foot care in diabetes. Diabetologia.

[12] Schaper N.C., Van Netten J.J., Apelqvist J., Lipsky B.A., Bakker K., International Working Group on the Diabetic Foot (2016). Prevention and management of foot problems in diabetes: A Summary Guidance for Daily Practice 2015, based on the IWGDF Guidance Documents. Diabetes Metab. Res. Rev..

[13] Kaminski M.R., Raspovic A., McMahon L.P., Lambert K.A., Erbas B., Mount P.F., Kerr P.G., Landorf K.B. (2017). Factors associated with foot ulceration and amputation in adults on dialysis: A cross-sectional observational study. BMC Nephrol..

[14] Kaminski M.R., Raspovic A., McMahon L.P., Strippoli G.F.M., Palmer S.C., Ruospo M., Dallimore S., Landorf K.B. (2015). Risk factors for foot ulceration and lower extremity amputation in adults with end-stage renal disease on dialysis: A systematic review and meta-analysis. Nephrol. Dial. Transplant..

[15] Robinson B.M., Akizawa T., Jager K.J., Kerr P.G., Saran R., Pisoni R.L. (2016). Factors affecting outcomes in patients reaching end-stage kidney disease worldwide: Differences in access to renal replacement therapy, modality use, and haemodialysis practices. Lancet.

[16] Moxey P.W., Gogalniceanu P., Hinchliffe R.J., Loftus I.M., Jones K.J., Thompson M.M., Holt P.J. (2011). Lower extremity amputations--a review of global variability in incidence. Diabet. Med..

[17] Kim H.K., Song S.O., Noh J., Jeong I.K., Lee B.W. (2020). Data configuration and publication trends for the Korean national health insurance and health insurance review & assessment database. Diabetes Metab. J..

[18] Yang H., Chen Y.-H., Hsieh T.-F., Chuang S.-Y., Wu M.-J. (2016). Prediction of mortality in incident hemodialysis patients: A validation and comparison of CHADS2, CHA2DS2, and CCI scores. PLoS ONE.

[19] King R.W., Malas M.B., Brothers T.E. (2019). Outcomes for peripheral vascular intervention and lower extremity bypass in kidney transplant recipients are superior to outcomes of patients remaining on dialysis. J. Vasc. Surg..

[20] Aitken E., Ramjug S., Buist L., Kingsmore D. (2012). The prognostic significance of iliac vessel calcification in renal transplantation. Transplant. Proc..

[21] Block G.A., Hulbert-Shearon T.E., Levin N.W., Port F.K. (1998). Association of serum phosphorus and calcium x phosphate product with mortality risk in chronic hemodialysis patients: A national study. Am. J. Kidney Dis..

[22] Graziani L., Silvestro A., Bertone V., Manara E., Alicandri A., Parrinello G., Manganoni A. (2007). Percutaneous transluminal angioplasty is feasible and effective in patients on chronic dialysis with severe peripheral artery disease. Nephrol. Dial. Transplant..

[23] Baghdasaryan P.A., Bae J.H., Yu W., Rowe V., Armstrong D.G., Shavelle D.M., Clavijo L.C. (2020). “The Renal Foot”-angiographic pattern of patients with chronic limb threatening ischemia and end-stage renal disease. Cardiovasc. Revasc. Med..

[24] Smilowitz N.R., Bhandari N., Berger J.S. (2020). Chronic kidney disease and outcomes of lower extremity revascularization for peripheral artery disease. Atherosclerosis.

[25] Vierthaler L., Callas P.W., Goodney P.P., Schanzer A., Patel V.I., Cronenwett J., Bertges D.J., Vascular Study Group of New England (2015). Determinants of survival and major amputation after peripheral endovascular intervention for critical limb ischemia. J. Vasc. Surg..

[26] Patzer R.E., Basu M., Larsen C.P., Pastan S.O., Mohan S., Patzer M., Konomos M., McClellan W.M., Lea J., Howard D. (2016). iChoose kidney: A clinical decision aid for kidney transplantation vs. dialysis treatment. Transplantation.

[27] Wolf M., Weir M.R., Kopyt N., Mannon R.B., Von Visger J., Deng H., Yue S., Vincenti F. (2016). A prospective cohort study of mineral metabolism after kidney transplantation. Transplantation.

[28] Long C.A., Mulder H., Fowkes F.G.R., Baumgartner I., Berger J.S., Katona B.G., Mahaffey K.W., Norgren L., Blomster J.I., Rockhold F.W. (2020). Incidence and factors associated with major amputation in patients with peripheral artery disease: Insights from the EUCLID trial. Circ. Cardiovasc. Qual. Outcomes.

[29] Laclaustra M., Casasnovas J.A., Fernández-Ortiz A., Fuster V., León-Latre M., Jiménez-Borreguero L.J., Pocovi M., Hurtado-Roca Y., Ordovas J.M., Jarauta E. (2016). Femoral and carotid subclinical atherosclerosis association with risk factors and coronary calcium: The AWHS study. J. Am. Coll. Cardiol..

[30] Lin C.-W., Armstrong D.G., Huang C.-H., Lin C.-H., Hung S.-Y., Liu P.-H., Huang Y.-Y. (2022). Diabetic foot disease in subjects with end-stage renal disease: A nationwide study over 14 years highlighting an emerging threat. Diabetes Res. Clin. Pract..

[31] Lüders F., Bunzemeier H., Engelbertz C., Malyar N.M., Meyborg M., Roeder N., Berger K., Reinecke H. (2016). CKD and acute and long-term outcome of patients with peripheral artery disease and critical limb ischemia. Clin. J. Am. Soc. Nephrol..

[32] Beckman J.A., Duncan M.S., Damrauer S.M., Wells Q.S., Barnett J.V., Wasserman D.H., Bedimo R.J., Butt A.A., Marconi V.C., Sico J.J. (2019). Microvascular disease, peripheral artery disease, and amputation. Circulation.

[33] Zubair M., Malik A., Ahmad J. (2015). Diabetic foot ulcer: A review. Am. J. Intern. Med..

[34] Charnogursky G.A., Emanuele N.V., Emanuele M.A. (2014). Neurologic complications of diabetes. Curr. Neurol. Neurosci. Rep..

[35] Lo H.-Y., Lin Y.-S., Lin D.S.-H., Lee J.-K., Chen W.-J. (2022). Association of statin therapy with major adverse cardiovascular and limb outcomes in patients with end-stage kidney disease and peripheral artery disease receiving maintenance dialysis. JAMA Netw. Open.

[36] Hsu C.-Y., Chen Y.-T., Su Y.-W., Chang C.-C., Huang P.-H., Lin S.-J. (2017). Statin therapy reduces future risk of lower-limb amputation in patients with diabetes and peripheral artery disease. J. Clin. Endocrinol. Metab..

[37] Sayiner Z.A., Can F.I., Akarsu E. (2019). Patients’ clinical charecteristics and predictors for diabetic foot amputation. Prim. Care Diabetes.

[38] Ahmad N., Thomas G.N., Gill P., Chan C., Torella F. (2014). Lower limb amputation in England: Prevalence, regional variation and relationship with revascularisation, deprivation and risk factors. A retrospective review of hospital data. J. R. Soc. Med..

[39] Jaar B.G., Astor B.C., Berns J.S., Powe N.R. (2004). Predictors of amputation and survival following lower extremity revascularization in hemodialysis patients. Kidney Int..

[40] Seeman T., Thomas D., Merkin S.S., Moore K., Watson K., Karlamangla A. (2018). The Great Recession worsened blood pressure and blood glucose levels in American adults. Proc. Natl. Acad. Sci. USA.

[41] Fanaroff A.C., Yang L., Nathan A.S., Khatana S.A.M., Julien H., Wang T.Y., Armstrong E.J., Treat-Jacobson D., Glaser J.D., Wang G. (2021). Geographic and socioeconomic disparities in major lower extremity amputation rates in metropolitan areas. J. Am. Heart Assoc..

[42] Stevens C.D., Schriger D.L., Raffetto B., Davis A.C., Zingmond D., Roby D.H. (2014). Geographic clustering of diabetic lower-extremity amputations in low-income regions of California. Health Aff..

[43] Cao J., Sharath S.E., Zamani N., Barshes N.R. (2019). Health care resource distribution of Texas counties with high rates of leg amputations. J. Surg. Res..

[44] McGinigle K.L., Kalbaugh C.A., Marston W.A. (2014). Living in a medically underserved county is an independent risk factor for major limb amputation. J. Vasc. Surg..

