# Supplementary material for: Risk Factors for Lower Extremity Amputation in Patients with End-Stage Kidney Disease: A Nationwide Cohort Study
Source: J Clin Med. 2023 Aug 30;12(17):5641. doi: 10.3390/jcm12175641 (PMC10488883; doi:10.3390/jcm12175641)
Supplement: Supplementary file 1 [file jcm-12-05641-s001.zip › jcm-2485669-supplementary.pdf]

Supplementary Table S1. Definition of Variables

Variable	Code Classification	Code in Detail
Acquired Immune Deficiency	ICD	B20, B21, B22, B24
Bypass surgery	practice code	O0163, O0164, O0165, O0166, O0167, O0168, O0169, O0170, O1645, O1646, O2064, O2067, O2065, O2068
CAD	ICD	I20, I21, I22, I23, I24, I25
Cancer	ICD	C00, C01, C02, C03, C04, C05, C06, C07, C08, C09, C10, C11, C12, C13, C14, C15, C16, C17, C18, C19, C20, C21, C22, C23, C24, C25, C26, C30, C31, C32, C33, C34, C37, C38, C39, C40, C41, C43, C44, C45, C46, C47, C48, C49, C50, C51, C52, C53, C54, C55, C56, C57, C58, C60, C61, C62, C63, C64, C65, C66, C67, C68, C69, C70, C71, C72, C73, C74, C75, C76, C77, C78, C79, C80, C81, C82, C83, C84, C85, C86, C88, C90, C91, C92, C93, C94, C95, C96, C97
CABG	practice code	O164, O183, OA64
Chronic Lung Disease	ICD	I278, I279, J40, J41, J42, J43, J44, J45, J46, J47, J60, J61, J62, J63, J64, J65, J66, J67, J684, J701, J703
Connective Tissue Disease	ICD	M05, M06, M315, M32, M33, M34, M351, M353, M360
CVD	ICD	G45, G46, H340, I60, I61, I62, I63, I64, I65, I66, I67, I68, I69
Diabetes without Complications	ICD	E100, E101, E106, E108, E109, E110, E111, E116, E118, E119, E120, E121, E126, E128, E129, E130, E131, E136, E138, E139, E140, E141, E146, E148, E149
Diabetes without Complications	ICD	E100, E101, E106, E108, E109, E110, E111, E116, E118, E119, E120, E121, E126, E128, E129, E130, E131, E136, E138, E139, E140, E141, E146, E148, E149
DM	ICD	E10, E11, E12, E13, E14
Dementia	ICD	F00, F01, F02, F03, F051, G30, G311
Depression	ICD	F32, F33
Dyslipidemia	ICD	E78
Endovascular procedure	practice code	M6597, M6605, M6613, M6620, HA858, HE323
Hematologic Malignancy	ICD	C00, C01, C02, C03, C04, C05, C06, C07, C08, C09, C10, C11, C12, C13, C14, C15, C16, C17, C18, C19, C20, C21, C22, C23, C24, C25, C26, C27, C28, C29, C30, C31, C32, C33, C34, C35, C36, C37, C38, C39, C40, C41, C42, C43, C44, C45, C46, C47, C48, C49, C50, C51, C52, C53, C54, C55, C56, C57, C58, C59, C60, C61, C62, C63, C64, C65, C66, C67,

		C68, C69, C70, C71, C72, C73, C74, C75, C76, C81, C82, C83, C84, C85, C86, C87, C88, C89, C90, C91, C92, C93, C94, C95, C96, C97, C98, C99
Hemiplegia	ICD	G041, G114, G801, G802, G81, G82, G830, G831, G832, G833, G834, G839
Hemorrhagic Stroke	ICD	I60, I61, I62
Hypertension	ICD	I10, I11
Ischemic Stroke	ICD	I63, I64
LER	practice code	HA858, HE323, M6597, M6605
MACE	ICD and/or practice code	(PCI, CABG, MI, Ischemic Stroke, and/or Hemorrhagic Stroke)
Metastatic Solid Tumor	ICD	C77, C78, C79, C80
MI	ICD	I21, I22, I23
Mild Liver Disease	ICD	B18, K700, K701, K702, K703, K709, K713, K714, K715, K717, K73, K74, K760, K762, K763, K764, K768, K769, Z944
Moderate to Severe Liver Disease	ICD	I850, I859, I864, I982, K704, K711, K721, K729, K765, K766, K767
MVD	ICD	(PND and/or Retinopathy)
PAD	ICD and/or practice code	(PVD and/or LER)
PCI	practice code	M655, M656, M657, M663
Peptic Ulcer Disease	ICD	K25, K26, K27, K28
PND	ICD	G62, G63, G64
PVD	ICD	I70, I71, I731, I738, I739, I771, I790, I792, K551, K558, K559, Z958, Z959
Retinopathy	ICD	E1031, E1032, E1033, E1131, E1132, E1133, E1231, E1232, E1233, E1331, E1332, E1333, E1431, E1432, E1433, H30, H31, H32, H33, H34, H35, H36

CABG = Coronary Artery Bypass Graft, CAD = Coronary Artery Disease, CPD = Chronic Pulmonary Disease, CVD = Cerebrovascular Disease, DM = Diabetes Mellitus, ICD = International Classification of Diseases, LER = Lower Extremity Revascularization, MACE = Major Adverse Cardiovascular Events, MI = Myocardial Infarction, MVD = Microvascular Disease, PAD = Peripheral Artery Disease, PCI = Percutaneous Coronary Intervention, PND = Peripheral Nerve Disease, PVD = Peripheral Vascular Disease

Supplementary Table S2. Charlson's Comorbidity Index

		Control (N = 214,490)	LEA (Total) (N = 6,348)	LEA (Total) AK (N = 363)	BK (N = 2,113)	FT (N = 3,872)	p-value*	p-value**
		N (%)	N (%)	N (%)	N (%)	N (%)		
CCI	Myocardial Infarction	63,419 (29.6)	2,430 (38.3)	136 (37.5)	788 (37.3)	1,506 (38.9)	<.0001	0.4511
	Congestive Heart Failure	54,941 (25.6)	1,880 (29.6)	109 (30.0)	589 (27.9)	1,182 (30.5)	<.0001	0.0981
	Peripheral Vascular Disease	18,700 (8.7)	908 (14.3)	70 (19.3)	286 (13.5)	552 (14.3)	<.0001	0.0152
	Cerebrovascular Disease	44,785 (20.9)	1,505 (23.7)	113 (31.1)	496 (23.5)	896 (23.1)	<.0001	0.0027
	Dementia	15,048 (7.0)	234 (3.7)	28 (7.7)	64 (3.0)	142 (3.7)	<.0001	<.0001
	Chronic Pulmonary Disease	62,538 (29.2)	1,654 (26.1)	89 (24.5)	526 (24.9)	1,039 (26.8)	<.0001	0.2077
	Rheumatologic Disease	10,567 (4.9)	185 (2.9)	14 (3.9)	59 (2.8)	112 (2.9)	<.0001	0.5333
	Peptic Ulcer Disease	59,618 (27.8)	1,749 (27.6)	98 (27.0)	610 (28.9)	1,041 (26.9)	0.6698	0.2523
	Mild Liver Disease	58,563 (27.3)	1,508 (23.8)	87 (24.0)	461 (21.8)	960 (24.8)	<.0001	0.0352
	Diabetes without Chronic Complications	31,184 (14.5)	345 (5.4)	17 (4.7)	111 (5.3)	217 (5.6)	<.0001	0.6869
	Diabetes with Chronic Complications	101,575 (47.4)	5,435 (85.6)	292 (80.4)	1,825 (86.4)	3,318 (85.7)	<.0001	0.0117
	Hemiplegia or Paraplegia	6,454 (3.0)	229 (3.6)	36 (9.9)	83 (3.9)	110 (2.8)	0.0061	<.0001
	Any Malignancy Including Leukemia	22,655 (10.6)	300 (4.7)	13 (3.6)	100 (4.7)	187 (4.8)	<.0001	0.5630
	Moderate or Severe Liver Disease	4,640 (2.2)	70 (1.1)	5 (1.4)	26 (1.2)	39 (1.0)	<.0001	0.6405
	Metastatic Solid Tumor	2,923 (1.4)	24 (0.4)	2 (0.6)	8 (0.4)	14 (0.4)	<.0001	0.8538
	Acquired Immune Deficiency	120 (0.1)	1 (0.0)	0 (0.0)	0 (0.0)	1 (0.0)	0.2708 ***	1.0000 ***

* Comparison between non-amputation and amputation groups
** Comparison between AK, BK, and FT groups
*** Fisher's exact test

AK = Above Knee Amputation, BK = Below Knee Amputation, CCI = Charlson's Comorbidity Index, FT = Foot or Toe Amputation, LEA = Lower Extremity Amputation

Supplementary Table S3. Patient Characteristics (Subgroup)

	KT					
	Total	Control	LEA (Total)	LEA (Total)		
	(N = 23,323)	(N = 22,938)	(N = 385)	AK (N = 9)	BK (N = 75)	FT (N = 301)
	N (%)	N (%)	N (%)	N (%)	N (%)	N (%)
Age (Mean, SD)	47.41 (11.41)	47.31 (11.42)	53.25 (8.52)	52.11 (10.25)	54.85 (8.18)	52.89 (8.53)
Age						
20-29	1,775 (7.6)	1,771 (7.7)	4 (1.0)	0 (0.0)	0 (0.0)	4 (1.3)
30-39	4,182 (17.9)	4,163 (18.1)	19 (4.9)	1 (11.1)	2 (2.7)	16 (5.3)
40-49	6,520 (28.0)	6,428 (28.0)	92 (23.9)	2 (22.2)	16 (21.3)	74 (24.6)
50-59	7,404 (31.7)	7,229 (31.5)	175 (45.5)	4 (44.4)	31 (41.3)	140 (46.5)
60-69	3,155 (13.5)	3,066 (13.4)	89 (23.1)	2 (22.2)	25 (33.3)	62 (20.6)
>70	287 (1.2)	281 (1.2)	6 (1.6)	0 (0.0)	1 (1.3)	5 (1.7)
Sex						
Male	13,889 (59.6)	13,601 (59.3)	288 (74.8)	5 (55.6)	59 (78.7)	224 (74.4)
Female	9,434 (40.4)	9,337 (40.7)	97 (25.2)	4 (44.4)	16 (21.3)	77 (25.6)
Residence						
Metropolitan	16,573 (71.1)	16,302 (71.1)	271 (70.4)	7 (77.8)	51 (68.0)	213 (70.8)
Non-metropolitan	6,750 (28.9)	6,636 (28.9)	114 (29.6)	2 (22.2)	24 (32.0)	88 (29.2)
Income						
0†	2,980 (12.8)	2,908 (12.7)	72 (18.7)	2 (22.2)	15 (20.0)	55 (18.3)
1st	3,692 (15.8)	3,647 (15.9)	45 (11.7)	1 (11.1)	9 (12.0)	35 (11.6)
2nd	3,740 (16.0)	3,695 (16.1)	45 (11.7)	1 (11.1)	6 (8.0)	38 (12.6)
3rd	5,323 (22.8)	5,256 (22.9)	67 (17.4)	1 (11.1)	10 (13.3)	56 (18.6)
4th	7,588 (32.5)	7,432 (32.4)	156 (40.5)	4 (44.4)	35 (46.7)	117 (38.9)
Past History						

CAD	5,954 (25.5)	5,772 (25.2)	182 (47.3)	2 (22.2)	45 (60.0)	135 (44.9)
CVD	2,040 (8.7)	1,963 (8.6)	77 (20.0)	4 (44.4)	15 (20.0)	58 (19.3)
DM	13,525 (58.0)	13,157 (57.4)	368 (95.6)	9 (100.0)	70 (93.3)	289 (96.0)
Hypertension	20,396 (87.5)	20,043 (87.4)	353 (91.7)	9 (100.0)	70 (93.3)	274 (91.0)
Dyslipidemia	15,436 (66.2)	15,180 (66.2)	256 (66.5)	4 (44.4)	48 (64.0)	204 (67.8)
CCI score (Mean, SD)	4.33 (1.68)	4.32 (1.67)	5.24 (1.62)	5.22 (1.64)	5.43 (1.69)	5.19 (1.61)
CCI ≥ 5	9,397 (40.3)	9,153 (39.9)	244 (63.4)	6 (66.7)	48 (64.0)	190 (63.1)
MVD	7,879 (33.8)	7,581 (33.0)	298 (77.4)	6 (66.7)	61 (81.3)	231 (76.7)
PND	2,438 (10.5)	2,310 (10.1)	128 (33.2)	2 (22.2)	25 (33.3)	101 (33.6)
Retinopathy	6,925 (29.7)	6,650 (29.0)	275 (71.4)	6 (66.7)	58 (77.3)	211 (70.1)
CPD	5,653 (24.2)	5,561 (24.2)	92 (23.9)	1 (11.1)	19 (25.3)	72 (23.9)
Cancer	1,822 (7.8)	1,802 (7.9)	20 (5.2)	0 (0.0)	2 (2.7)	18 (6.0)
Depression	2,004 (8.6)	1,934 (8.4)	70 (18.2)	2 (22.2)	16 (21.3)	52 (17.3)
PAD	4,573 (19.6)	4,428 (19.3)	145 (37.7)	3 (33.3)	25 (33.3)	117 (38.9)
PVD	3,413 (14.6)	3,305 (14.4)	108 (28.1)	2 (22.2)	21 (28.0)	85 (28.2)
LER	1,634 (7.0)	1,574 (6.9)	60 (15.6)	2 (22.2)	11 (14.7)	47 (15.6)
MACE	2,011 (8.6)	1,923 (8.4)	88 (22.9)	4 (44.4)	19 (25.3)	65 (21.6)
PCI	538 (2.3)	508 (2.2)	30 (7.8)	0 (0.0)	10 (13.3)	20 (6.6)
CABG	131 (0.6)	125 (0.5)	6 (1.6)	0 (0.0)	2 (2.7)	4 (1.3)
MI	492 (2.1)	472 (2.1)	20 (5.2)	0 (0.0)	6 (8.0)	14 (4.7)
Ischemic Stroke	915 (3.9)	863 (3.8)	52 (13.5)	4 (44.4)	11 (14.7)	37 (12.3)
Hemorrhagic Stroke	232 (1.0)	231 (1.0)	1 (0.3)	0 (0.0)	0 (0.0)	1 (0.3)
Medication						
RAS inhibitor	17,953 (77.0)	17,639 (76.9)	314 (81.6)	8 (88.9)	62 (82.7)	244 (81.1)

Antiplatelet	10,080 (43.2)	9,796 (42.7)	284 (73.8)	7 (77.8)	61 (81.3)	216 (71.8)
Anticoagulant	373 (1.6)	361 (1.6)	12 (3.1)	0 (0.0)	3 (4.0)	9 (3.0)
Surgery						
Endovascular	1,214 (5.2)	1,050 (4.6)	164 (42.6)	6 (66.7)	47 (62.7)	111 (36.9)
Bypass	27 (0.1)	17 (0.1)	10 (2.6)	1 (11.1)	4 (5.3)	5 (1.7)
All cause death	1,839 (7.9)	1,659 (7.2)	90 (23.4)	6 (66.7)	27 (36.0)	57 (18.9)
CV mortality	282 (1.2)	248 (1.1)	17 (4.4)	2 (22.2)	3 (4.0)	12 (4.0)

	Dialysis					
	Total	Control	LEA (Total)	LEA (Total)		
	(N = 197,515)	(N = 191,552)	(N = 5,963)	AK (N = 354)	BK (N = 2,038)	FT (N = 3,571)
	N (%)	N (%)	N (%)	N (%)	N (%)	N (%)
Age (Mean, SD)	63.00 (14.34)	63.12 (14.41)	59.24 (11.14)	58.98 (10.52)	58.18 (10.65)	59.87 (11.43)
Age						
20-29	3,368 (1.7)	3,345 (1.7)	23 (0.4)	1 (0.3)	13 (0.6)	9 (0.3)
30-39	9,651 (4.9)	9,457 (4.9)	194 (3.3)	10 (2.8)	65 (3.2)	119 (3.3)
40-49	23,381 (11.8)	22,394 (11.7)	987 (16.6)	61 (17.2)	349 (17.1)	577 (16.2)
50-59	38,929 (19.7)	37,131 (19.4)	1,798 (30.2)	111 (31.4)	670 (32.9)	1,017 (28.5)
60-69	48,838 (24.7)	47,023 (24.5)	1,815 (30.4)	109 (30.8)	654 (32.1)	1,052 (29.5)
>70	73,348 (37.1)	72,202 (37.7)	1,146 (19.2)	62 (17.5)	287 (14.1)	797 (22.3)
Sex						
Male	113,945 (57.7)	109,734 (57.3)	4,211 (70.6)	236 (66.7)	1,456 (71.4)	2,519 (70.5)
Female	83,570 (42.3)	81,818 (42.7)	1,752 (29.4)	118 (33.3)	582 (28.6)	1,052 (29.5)
Residence						
Metropolitan	131,854 (66.8)	127,701 (66.7)	4,153 (69.6)	238 (67.2)	1,425 (69.9)	2,490 (69.7)
Non-metropolitan	65,661 (33.2)	63,851 (33.3)	1,810 (30.4)	116 (32.8)	613 (30.1)	1,081 (30.3)

Income						
0†	33,885 (17.2)	32,772 (17.1)	1,113 (18.7)	83 (23.4)	414 (20.3)	616 (17.3)
1st	32,491 (16.4)	31,513 (16.5)	978 (16.4)	65 (18.4)	331 (16.2)	582 (16.3)
2nd	32,260 (16.3)	31,254 (16.3)	1,006 (16.9)	62 (17.5)	336 (16.5)	608 (17.0)
3rd	40,240 (20.4)	38,984 (20.4)	1,256 (21.1)	72 (20.3)	429 (21.1)	755 (21.1)
4th	58,639 (29.7)	57,029 (29.8)	1,610 (27.0)	72 (20.3)	528 (25.9)	1,010 (28.3)
Past History						
CAD	59,895 (30.3)	57,647 (30.1)	2,248 (37.7)	134 (37.9)	743 (36.5)	1,371 (38.4)
CVD	44,250 (22.4)	42,822 (22.4)	1,428 (23.9)	109 (30.8)	481 (23.6)	838 (23.5)
DM	127,872 (64.7)	122,389 (63.9)	5,483 (92.0)	302 (85.3)	1,890 (92.7)	3,291 (92.2)
Hypertension	164,197 (83.1)	158,913 (83.0)	5,284 (88.6)	308 (87.0)	1,790 (87.8)	3,186 (89.2)
Dyslipidemia	115,968 (58.7)	112,155 (58.6)	3,813 (63.9)	217 (61.3)	1,276 (62.6)	2,320 (65.0)
CCI score (Mean, SD)	4.92 (2.09)	4.91 (2.10)	5.02 (1.82)	5.25 (1.64)	4.97 (1.69)	5.03 (1.61)
CCI ≥ 5	103,921 (52.6)	100,526 (52.5)	3,395 (56.9)	217 (61.3)	1,125 (55.2)	2,053 (57.5)
MVD	78,015 (39.5)	73,992 (38.6)	4,023 (67.5)	219 (61.9)	1,401 (68.7)	2,403 (67.3)
PND	31,875 (16.1)	29,954 (15.6)	1,921 (32.2)	112 (31.6)	685 (33.6)	1,124 (31.5)
Retinopathy	64,871 (32.8)	61,476 (32.1)	3,395 (56.9)	172 (48.6)	1,195 (58.6)	2,028 (56.8)
CPD	58,539 (29.6)	56,977 (29.7)	1,562 (26.2)	88 (24.9)	507 (24.9)	967 (27.1)
Cancer	21,335 (10.8)	21,052 (11.0)	283 (4.7)	14 (4.0)	98 (4.8)	171 (4.8)
Depression	20,281 (10.3)	19,736 (10.3)	545 (9.1)	30 (8.5)	182 (8.9)	333 (9.3)
PAD	41,538 (21.0)	39,776 (20.8)	1,762 (29.5)	121 (34.2)	572 (28.1)	1,069 (29.9)
PVD	39,294 (19.9)	37,631 (19.6)	1,663 (27.9)	114 (32.2)	532 (26.1)	1,017 (28.5)
LER	4,031 (2.0)	3,735 (1.9)	296 (5.0)	21 (5.9)	97 (4.8)	178 (5.0)
MACE	41,566 (21.0)	40,039 (20.9)	1,527 (25.6)	107 (30.2)	554 (27.2)	866 (24.3)
PCI	6,880 (3.5)	6,523 (3.4)	357 (6.0)	24 (6.8)	123 (6.0)	210 (5.9)
CABG	1,096 (0.6)	1,012 (0.5)	84 (1.4)	7 (2.0)	40 (2.0)	37 (1.0)

MI	11,133 (5.6)	10,685 (5.6)	448 (7.5)	28 (7.9)	164 (8.0)	256 (7.2)
Ischemic Stroke	27,224 (13.8)	26,278 (13.7)	946 (15.9)	69 (19.5)	343 (16.8)	534 (15.0)
Hemorrhagic Stroke	3,933 (2.0)	3,861 (2.0)	72 (1.2)	9 (2.5)	19 (0.9)	44 (1.2)
Medication						
RAS inhibitor	137,896 (69.8)	133,097 (69.5)	4,799 (80.5)	281 (79.4)	1,631 (80.0)	2,887 (80.8)
Antiplatelet	96,425 (48.8)	92,771 (48.4)	3,654 (61.3)	223 (63.0)	1,199 (58.8)	2,232 (62.5)
Anticoagulant	7,079 (3.6)	6,864 (3.6)	215 (3.6)	14 (4.0)	68 (3.3)	133 (3.7)
Surgery						
Endovascular	55,972 (28.3)	53,049 (27.7)	2,923 (49.0)	175 (49.4)	1,049 (51.5)	1,699 (47.6)
Bypass	275 (0.1)	187 (0.1)	88 (1.5)	17 (4.8)	40 (2.0)	31 (0.9)
All cause death	107,380 (54.4)	99,070 (51.7)	4,155 (69.7)	297 (83.9)	1,589 (78.0)	2,269 (63.5)
CV mortality	19,702 (10.0)	18,638 (9.7)	532 (8.9)	39 (11.0)	187 (9.2)	306 (8.6)

* Comparison between non-amputation and amputation groups
** Comparison between AK, BK, and FT groups
† Beneficiaries of National Basic Livelihood

AK = Above Knee Amputation, BK = Below Knee Amputation, CABG = Coronary Artery Bypass Graft, CAD = Coronary Artery Disease, CCI = Charlson's Comorbidity Index, CPD = Chronic Pulmonary Disease, CV = Cardiovascular, CVD = Cerebrovascular Disease, DM = Diabetes Mellitus, FT = Foot or Toe Amputation, KT = Kidney Transplantation, LEA = Lower Extremity Amputation, LER = Lower Extremity Revascularization, MACE = Major Adverse Cardiovascular Events, MI = Myocardial Infarction, MVD = Microvascular Disease, PAD = Peripheral Artery Disease, PCI = Percutaneous Coronary Intervention, PND = Peripheral Nerve Disease, PVD = Peripheral Vascular Disease, RAS = Renin-Angiotensin System, SD = Standard Deviation

Supplementary Table S4. Charlson's Comorbidity Index (Subgroup)

		KT				
		Control	LEA (Total)	LEA (Total)		
		(N = 22,938)	(N = 385)	AK (N = 9)	BK (N = 75)	FT (N = 301)
		N (%)	N (%)	N (%)	N (%)	N (%)
CCI	Myocardial Infarction	5,772 (25.2)	182 (47.3)	2 (22.2)	45 (60.0)	135 (44.9)
	Congestive Heart Failure	4,377 (19.1)	114 (29.6)	3 (33.3)	23 (30.7)	88 (29.2)
	Peripheral Vascular Disease	1,496 (6.5)	56 (14.5)	0 (0.0)	15 (20.0)	41 (13.6)
	Cerebrovascular Disease	1,963 (8.6)	77 (20.0)	4 (44.4)	15 (20.0)	58 (19.3)
	Dementia	113 (0.5)	4 (1.0)	1 (11.1)	0 (0.0)	3 (1.0)
	Chronic Pulmonary Disease	5,561 (24.2)	92 (23.9)	1 (11.1)	19 (25.3)	72 (23.9)
	Rheumatologic Disease	1,017 (4.4)	12 (3.1)	0 (0.0)	2 (2.7)	10 (3.3)
	Peptic Ulcer Disease	7,503 (32.7)	135 (35.1)	1 (11.1)	32 (42.7)	102 (33.9)
	Mild Liver Disease	7,384 (32.2)	133 (34.5)	4 (44.4)	30 (40.0)	99 (32.9)
	Diabetes without Chronic Complications	4,748 (20.7)	23 (6.0)	1 (11.1)	6 (8.0)	16 (5.3)
	Diabetes with Chronic Complications	8,256 (36.0)	345 (89.6)	8 (88.9)	64 (85.3)	273 (90.7)
	Hemiplegia or Paraplegia	109 (0.5)	10 (2.6)	2 (22.2)	1 (1.3)	7 (2.3)
	Any Malignancy Including Leukemia	1,787 (7.8)	19 (4.9)	0 (0.0)	2 (2.7)	17 (5.6)
	Moderate or Severe Liver Disease	212 (0.9)	3 (0.8)	0 (0.0)	0 (0.0)	3 (1.0)
	Metastatic Solid Tumor	70 (0.3)	1 (0.3)	0 (0.0)	0 (0.0)	1 (0.3)
	Acquired Immune Deficiency	28 (0.1)	0 (0.0)	0 (0.0)	0 (0.0)	0 (0.0)

		Dialysis				
		Control	LEA (Total)	LEA (Total)		
		(N = 191,552)	(N = 5,963)	AK (N = 354)	BK (N = 2,038)	FT (N = 3,571)
		N(%)	N(%)	N(%)	N(%)	N(%)
CCI	Myocardial Infarction	57,647 (30.1)	2,248 (37.7)	134 (37.9)	743 (36.5)	1,371 (38.4)
	Congestive Heart Failure	50,564 (26.4)	1,766 (29.6)	106 (29.9)	566 (27.8)	1,094 (30.6)
	Peripheral Vascular Disease	17,204 (9.0)	852 (14.3)	70 (19.8)	271 (13.3)	511 (14.3)
	Cerebrovascular Disease	42,822 (22.4)	1,428 (23.9)	109 (30.8)	481 (23.6)	838 (23.5)
	Dementia	14,935 (7.8)	230 (3.9)	27 (7.6)	64 (3.1)	139 (3.9)
	Chronic Pulmonary Disease	56,977 (29.7)	1,562 (26.2)	88 (24.9)	507 (24.9)	967 (27.1)
	Rheumatologic Disease	9,550 (5.0)	173 (2.9)	14 (4.0)	57 (2.8)	102 (2.9)
	Peptic Ulcer Disease	52,115 (27.2)	1,614 (27.1)	97 (27.4)	578 (28.4)	939 (26.3)
	Mild Liver Disease	51,179 (26.7)	1,375 (23.1)	83 (23.4)	431 (21.1)	861 (24.1)
	Diabetes without Chronic Complications	26,436 (13.8)	322 (5.4)	16 (4.5)	105 (5.2)	201 (5.6)
	Diabetes with Chronic Complications	93,319 (48.7)	5,090 (85.4)	284 (80.2)	1,761 (86.4)	3,045 (85.3)
	Hemiplegia or Paraplegia	6,345 (3.3)	219 (3.7)	34 (9.6)	82 (4.0)	103 (2.9)
	Any Malignancy Including Leukemia	20,868 (10.9)	281 (4.7)	13 (3.7)	98 (4.8)	170 (4.8)
	Moderate or Severe Liver Disease	4,428 (2.3)	67 (1.1)	5 (1.4)	26 (1.3)	36 (1.0)
	Metastatic Solid Tumor	2,853 (1.5)	23 (0.4)	2 (0.6)	8 (0.4)	13 (0.4)
	Acquired Immune Deficiency	92 (0.0)	1 (0.0)	0 (0.0)	0 (0.0)	1 (0.0)

* Comparison between non-amputation and amputation groups
** Comparison between AK, BK, and FT groups

AK = Above Knee Amputation, BK = Below Knee Amputation, CCI = Charlson's Comorbidity Index, FT = Foot or Toe Amputation, KT = Kidney Transplantation, LEA = Lower Extremity Amputation

Supplementary Figure S1. Incidence of Lower Extremity Amputation

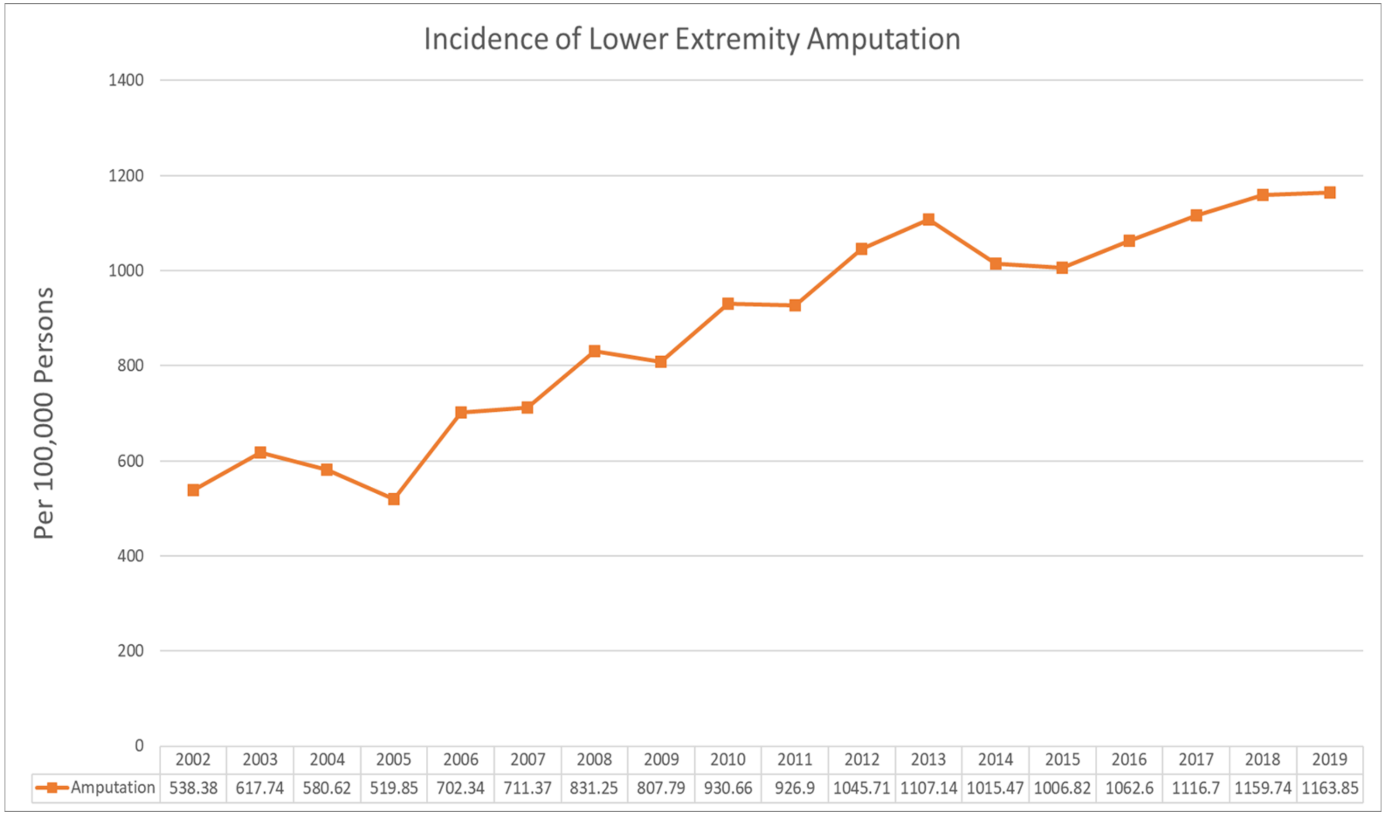

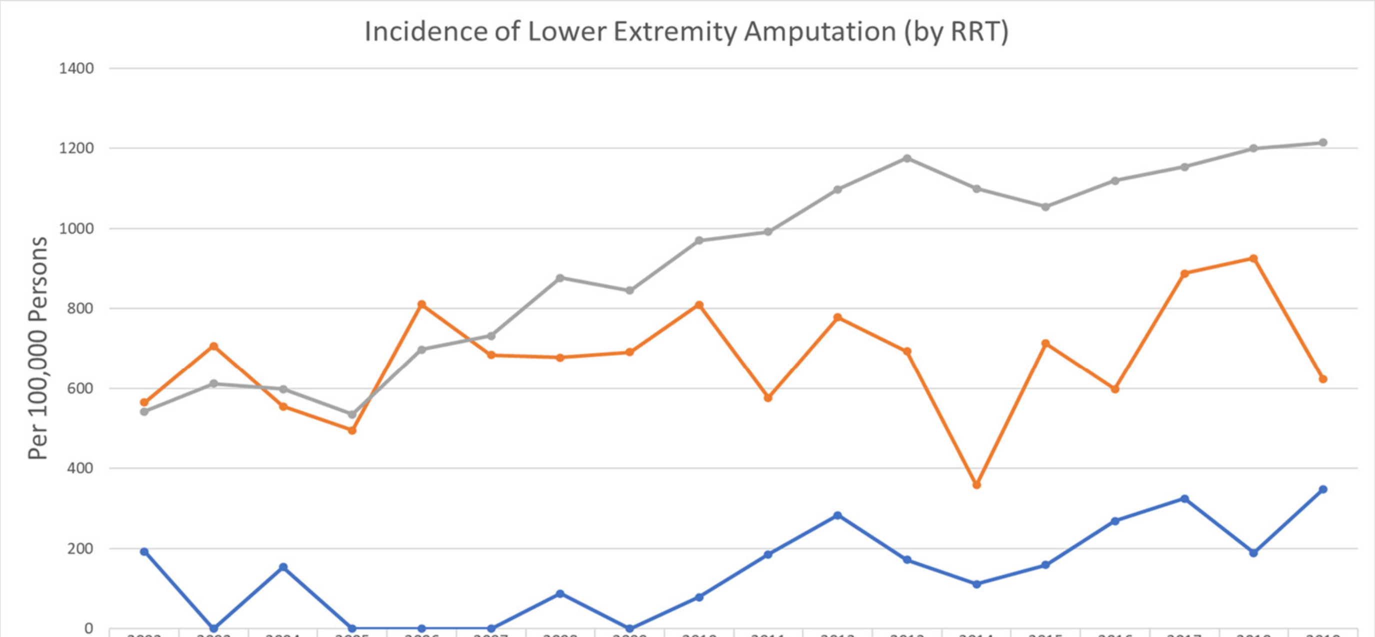

	2002	2003	2004	2005	2006	2007	2008	2009	2010	2011	2012	2013	2014	2015	2016	2017	2018	2019
KT	193.05	0	153.85	0	0	0	87.64	0	78.74	185.53	283.45	172.41	111.23	158.9	268.82	324.98	189.39	348.58
PD	564.74	707.29	554.92	495.53	811.36	684.65	678.11	691.94	809.84	576.37	778.56	693.43	358.49	713.05	598.56	888.32	926.34	623.05
HD	541.91	612.19	598.31	535.17	697.86	732.2	876.97	845.77	970.64	991.69	1098.1	1175.34	1099.68	1054.51	1120.03	1154.35	1199.99	1215.02

HD = Hemodialysis, KT = Kidney Transplantation, PD = Peritoneal Dialysis, RRT = Renal Replacement Therapy

Supplementary Table S5. Multivariable Cox Regression (Subgroup)

		Multivariable Cox Regression (Subgroup = KT)			
		LEA (Total)	AK	BK	FT
	Variables	HR (95% CI)	HR (95% CI)	HR (95% CI)	HR (95% CI)
Past History	DM	2.08 (1.68,2.59)	1.36 (1.05,1.75)	1.42 (1.11,1.81)	2.02 (1.62,2.51)
	Hypertension	1.03 (0.80,1.33)	1.06 (0.75,1.49)	1.05 (0.76,1.45)	1.02 (0.79,1.33)
	Dyslipidemia	0.90 (0.77,1.06)	0.79 (0.61,0.97)	0.81 (0.66,1.00	0.90 (0.76,1.07)
	CPD	0.92 (0.77,1.11)	0.96 (0.73,1.26)	0.95 (0.75,1.22)	0.93 (0.77,1.13)
	Cancer	0.73 (0.52,1.01)	0.80 (0.50,1.27)	0.72 (0.47,1.12)	0.77 (0.55,1.07)
	MACE	0.84 (0.67,1.05)	0.44 (0.27,0.73)	0.58 (0.39,0.84)	0.80 (0.62,1.02)
	PAD	1.27 (1.06,1.51)	0.91 (0.67,1.22)	0.96 (0.74,1.24)	1.27 (1.06,1.54)
	MVD	2.24 (1.90,2.64)	1.40 (1.11,1.77)	1.65 (1.33,2.04)	2.17 (1.83,2.58)

		Multivariable Cox Regression (Subgroup = Dialysis)			
		LEA (Total)	AK	BK	FT
	Variables	HR (95% CI)	HR (95% CI)	HR (95% CI)	HR (95% CI)
Past History	DM	1.65 (1.60, 1.70)	1.65 (1.59, 1.70)	1.42 (1.38, 1.47)	1.51 (1.46, 1.56)
	Hypertension	1.01 (0.97, 1.05)	1.01 (0.97, 1.05)	1.01 (0.97, 1.05)	1.01 (0.97, 1.05)
	Dyslipidemia	0.78 (0.76, 0.80)	0.78 (0.76, 0.80)	0.73 (0.71, 0.75)	0.74 (0.72, 0.76)
	CPD	0.96 (0.93, 0.98)	0.96 (0.93, 0.98)	0.95 (0.92, 0.98)	0.95 (0.92, 0.98)
	Cancer	0.79 (0.75, 0.83)	0.79 (0.75, 0.83)	0.81 (0.77, 0.85)	0.80 (0.77, 0.84)
	MACE	0.32 (0.30, 0.33)	0.32 (0.30, 0.33)	0.14 (0.13, 0.15)	0.20 (0.19, 0.22)
	PAD	1.03 (1.00, 1.06)	1.03 (1.00, 1.06)	0.95 (0.92, 0.98)	0.97 (0.94, 1.00)
	MVD	1.35 (1.32, 1.39)	1.35 (1.32, 1.39)	1.23 (1.19, 1.27)	1.28 (1.24, 1.32)

AK = Above Knee Amputation, BK = Below Knee Amputation, CI = Confidence Interval, CPD = Chronic Pulmonary Disease, DM = Diabetes Mellitus, FT = Foot or Toe

Amputation, HR = Hazard Ratio, KT = Kidney Transplantation, LEA = Lower Extremity Amputation, MACE = Major Adverse Cardiovascular Events, MVD = Microvascular Disease, PAD = Peripheral Artery Disease

Supplementary Table S6. Multivariable Cox Regression (CCI)

		Multivariable Cox Regression (Adjusted)			
		LEA (Total)	AK	BK	FT
	Variables	HR (95% CI)	HR (95% CI)	HR (95% CI)	HR (95% CI)
Age	20-29	0.13 (0.11, 0.15)	0.13 (0.11, 0.16)	0.13 (0.11, 0.16)	0.13 (0.11, 0.15)
	30-39	0.29 (0.27, 0.31)	0.28 (0.25, 0.30)	0.28 (0.25, 0.30)	0.29 (0.26, 0.31)
	40-49	0.64 (0.62, 0.67)	0.61 (0.58, 0.64)	0.62 (0.59, 0.65)	0.63 (0.61, 0.66)
	50-59	1	1	1	1
	60-69	1.52 (1.47, 1.57)	1.62 (1.56, 1.68)	1.58 (1.53, 1.64)	1.55 (1.50, 1.60)
	>70	1.93 (1.87, 1.99)	2.07 (2.00, 2.15)	2.00 (1.93, 2.07)	2.00 (1.94, 2.07)
Income	0[†]	1.58 (1.53, 1.63)	1.56 (1.50, 1.62)	1.57 (1.52, 1.63)	1.55 (1.50, 1.61)
	1	1.08 (1.04, 1.12)	1.07 (1.03, 1.11)	1.07 (1.03, 1.11)	1.07 (1.03, 1.11)
	2	1.10 (1.06, 1.14)	1.10 (1.06, 1.15)	1.10 (1.06, 1.14)	1.09 (1.05, 1.14)
	3	1.06 (1.03, 1.10)	1.07 (1.03, 1.11)	1.07 (1.03, 1.11)	1.06 (1.03, 1.10)
	4	1	1	1	1
Residence	Metropolitan	0.89 (0.87, 0.92)	0.87 (0.85, 0.90)	0.88 (0.86, 0.90)	0.89 (0.87, 0.91)
	Non-metropolitan	1	1	1	1
Sex	Male	1.22 (1.19, 1.25)	1.14 (1.11, 1.17)	1.17 (1.15, 1.20)	1.20 (1.17, 1.23)
	Female	1	1	1	1
ESKD (RRT)	HD	1	1	1	1
	PD	1.30 (1.25, 1.35)	1.28 (1.23, 1.34)	1.30 (1.24, 1.35)	1.28 (1.23, 1.33)
	KT	0.23 (0.21, 0.25)	0.14 (0.12, 0.15)	0.15 (0.14, 0.17)	0.22 (0.20, 0.24)
CCI Score	≥ 5	1.02 (0.98, 1.06)	1.02 (0.98, 1.07)	1.02 (0.97, 1.06)	1.02 (0.98, 1.07)
	< 5	1	1	1	1
Medication	Statin	0.67 (0.62, 0.73)	0.59 (0.54, 0.65)	0.62 (0.57, 0.67)	0.65 (0.60, 0.70)
	RAS inhibitor	0.95 (0.93, 0.98)	0.92 (0.90, 0.95)	0.94 (0.91, 0.96)	0.94 (0.92, 0.97)

	Antiplatelet	0.86 (0.83, 0.88)	0.78 (0.75, 0.80)	0.80 (0.78, 0.82)	0.83 (0.81, 0.86)
	Anticoagulant	1.00 (0.92, 1.08)	0.85 (0.78, 0.94)	0.90 (0.82, 0.98)	0.95 (0.87, 1.03)
Surgery	Endovascular	1.09 (1.06, 1.12)	1.05 (1.02, 1.08)	1.07 (1.05, 1.10)	1.07 (1.04, 1.10)
	Bypass	1.86 (1.58, 2.19)	1.67 (1.31, 2.14)	1.76 (1.43, 2.17)	1.59 (1.28, 1.98)
CCI	Myocardial Infarction	0.97 (0.95, 1.00)	0.95 (0.91, 0.98)	0.96 (0.93, 0.99)	0.96 (0.93, 0.99)
	Congestive Heart Failure	1.20 (1.17, 1.24)	1.18 (1.14, 1.22)	1.19 (1.15, 1.23)	1.20 (1.16, 1.24)
	Peripheral Vascular Disease	1.11 (1.07, 1.16)	1.03 (0.98, 1.08)	1.05 (1.00, 1.10)	1.08 (1.03, 1.13)
	Cerebrovascular Disease	0.49 (0.47, 0.51)	0.36 (0.34, 0.38)	0.40 (0.38, 0.42)	0.44 (0.42, 0.46)
	Dementia	0.77 (0.72, 0.82)	0.72 (0.67, 0.78)	0.72 (0.67, 0.78)	0.74 (0.69, 0.80)
	Chronic Pulmonary Disease	0.97 (0.94, 1.00)	0.95 (0.92, 0.98)	0.96 (0.93, 0.99)	0.96 (0.94, 0.99)
	Rheumatologic Disease	0.95 (0.89, 1.00)	0.96 (0.90, 1.02)	0.95 (0.90, 1.01)	0.95 (0.89, 1.01)
	Peptic Ulcer Disease	0.97 (0.94, 0.99)	0.98 (0.95, 1.01)	0.98 (0.95, 1.01)	0.96 (0.94, 0.99)
	Mild Liver Disease	0.89 (0.87, 0.92)	0.89 (0.86, 0.92)	0.89 (0.86, 0.91)	0.90 (0.87, 0.92)
	Diabetes without Chronic Complications	1.04 (0.99, 1.09)	0.92 (0.88, 0.97)	0.97 (0.92, 1.01)	1.00 (0.95, 1.04)
	Diabetes with Chronic Complications	1.99 (1.94, 2.05)	1.57 (1.53, 1.63)	1.72 (1.67, 1.78)	1.84 (1.78, 1.89)
	Hemiplegia or Paraplegia	0.85 (0.77, 0.94)	0.64 (0.56, 0.74)	0.70 (0.62, 0.80)	0.69 (0.61, 0.78)
	Any Malignancy Including Leukemia	0.82 (0.78, 0.86)	0.83 (0.78, 0.87)	0.83 (0.76, 0.87)	0.82 (0.78, 0.86)
	Moderate or Severe Liver Disease	1.06 (0.96, 1.16)	1.06 (0.95, 1.17)	1.06 (0.96, 1.18)	1.04 (0.94, 1.15)
	Metastatic Solid Tumor	1.18 (1.03, 1.34)	1.17 (1.02, 1.35)	1.17 (1.02, 1.34)	1.17 (1.02, 1.34)
	Acquired Immune Deficiency	0.37 (0.16, 0.9)	0.35 (0.13, 0.93)	0.34 (0.13, 0.9)	0.39 (0.16, 0.95)

* Comparison between non-amputation and amputation groups
** Comparison between AK, BK, and FT groups
† Beneficiaries of National Basic Livelihood

AK = Above Knee Amputation, BK = Below Knee Amputation, CCI = Charlson's Comorbidity Index, CI = Confidence Interval, ESKD = End, stage Kidney Disease, FT = Foot or Toe Amputation, HD = Hemodialysis, HR = Hazard Ratio, KT = Kidney Transplantation, LEA = Lower Extremity Amputation, PD = Peritoneal Dialysis, RAS = Renin-Angiotensin System, RRT = Renal Replacement Therapy

Supplementary Table S7. Multivariable Cox Regression (Subgroup)

		Multivariable Cox Regression (Subgroup = KT)			
		LEA (Total)	AK	BK	FT
	Variables	HR (95% CI)	HR (95% CI)	HR (95% CI)	HR (95% CI)
CCI	Myocardial Infarction	1.17 (0.98, 1.41)	1.24 (0.94, 1.63)	1.39 (1.09, 1.77)	1.15 (0.95, 1.40)
	Congestive Heart Failure	1.36 (1.13, 1.64)	1.19 (0.89, 1.60)	1.29 (0.99, 1.67)	1.30 (1.06, 1.59)
	Peripheral Vascular Disease	1.07 (0.83, 1.38)	0.78 (0.48, 1.26)	1.08 (0.74, 1.57)	1.01 (0.76, 1.34)
	Cerebrovascular Disease	0.82 (0.65, 1.04)	0.57 (0.36, 0.89)	0.65 (0.45, 0.94)	0.78 (0.61, 1.02)
	Dementia	2.03 (1.07, 3.84)	3.91 (1.79, 8.54)	2.73 (1.19, 6.23)	2.07 (1.05, 4.07)
	Chronic Pulmonary Disease	0.94 (0.78, 1.14)	1.00 (0.76, 1.32)	1.01 (0.79, 1.30)	0.95 (0.78, 1.16)
	Rheumatologic Disease	0.76 (0.50, 1.17)	0.79 (0.43, 1.46)	0.75 (0.43, 1.32)	0.79 (0.50, 1.23)
	Peptic Ulcer Disease	0.90 (0.76, 1.06)	1.12 (0.88, 1.43)	1.14 (0.91, 1.42)	0.90 (0.75, 1.08)
	Mild Liver Disease	1.07 (0.90, 1.27)	1.07 (0.83, 1.38)	1.12 (0.89, 1.42)	1.03 (0.86, 1.24)
	Diabetes without Chronic Complications	1.33 (0.99, 1.78)	1.13 (0.81, 1.59)	1.19 (0.86, 1.65)	1.26 (0.93, 1.71)
	Diabetes with Chronic Complications	3.61 (2.91, 4.47)	1.81 (1.39, 2.38)	2.09 (1.62, 2.69)	3.46 (2.78, 4.32)
	Hemiplegia or Paraplegia	2.70 (1.42, 5.16)	1.51 (0.36, 6.30)	0.69 (0.10, 5.02)	2.46 (1.14, 5.32)
	Any Malignancy Including Leukemia	0.83 (0.60, 1.17)	0.94 (0.58, 1.50)	0.88 (0.56, 1.37)	0.86 (0.61, 1.22)
	Moderate or Severe Liver Disease	0.84 (0.40, 1.78)	1.13 (0.41, 3.06)	0.98 (0.36, 2.65)	0.90 (0.42, 1.92)
	Metastatic Solid Tumor	0.84 (0.12, 6.09)	–	–	0.82 (0.11, 5.98)
	Acquired Immune Deficiency	–	–	–	–

	Multivariable Cox Regression (Subgroup = Dialysis)

	Variables	LEA (Total) HR (95% CI)	AK HR (95% CI)	BK HR (95% CI)	FT HR (95% CI)
CCI	Myocardial Infarction	0.98 (0.95, 1.01)	0.95 (0.92, 0.99)	0.96 (0.93, 1.00)	0.97 (0.94, 1.00)
	Congestive Heart Failure	1.19 (1.16, 1.23)	1.18 (1.14, 1.22)	1.18 (1.14, 1.22)	1.19 (1.15, 1.23)
	Peripheral Vascular Disease	1.10 (1.06, 1.15)	1.03 (0.98, 1.08)	1.04 (0.99, 1.09)	1.07 (1.02, 1.12)
	Cerebrovascular Disease	0.47 (0.45, 0.49)	0.35 (0.33, 0.37)	0.39 (0.37, 0.41)	0.42 (0.41, 0.44)
	Dementia	0.76 (0.71, 0.81)	0.71 (0.65, 0.76)	0.71 (0.66, 0.76)	0.73 (0.68, 0.79)
	Chronic Pulmonary Disease	0.97 (0.94, 1.00)	0.95 (0.92, 0.99)	0.96 (0.93, 0.99)	0.97 (0.94, 1.00)
	Rheumatologic Disease	0.93 (0.88, 0.99)	0.94 (0.88, 1.00)	0.94 (0.88, 1.00)	0.93 (0.88, 0.99)
	Peptic Ulcer Disease	1.01 (0.98, 1.03)	1.02 (0.99, 1.05)	1.02 (0.99, 1.05)	1.00 (0.97, 1.03)
	Mild Liver Disease	0.9 (0.87, 0.93)	0.90 (0.87, 0.93)	0.89 (0.86, 0.92)	0.90 (0.88, 0.93)
	Diabetes without Chronic Complications	1.11 (1.06, 1.17)	0.99 (0.94, 1.04)	1.04 (0.99, 1.09)	1.07 (1.02, 1.12)
	Diabetes with Chronic Complications	1.98 (1.92, 2.04)	1.58 (1.53, 1.63)	1.73 (1.67, 1.78)	1.82 (1.76, 1.88)
	Hemiplegia or Paraplegia	0.82 (0.73, 0.91)	0.62 (0.53, 0.71)	0.68 (0.60, 0.77)	0.66 (0.59, 0.75)
	Any Malignancy Including Leukemia	0.82 (0.78, 0.86)	0.83 (0.79, 0.88)	0.83 (0.79, 0.88)	0.83 (0.78, 0.87)
	Moderate or Severe Liver Disease	1.02 (0.93, 1.12)	1.01 (0.91, 1.12)	1.02 (0.93, 1.13)	1.00 (0.91, 1.11)
	Metastatic Solid Tumor	1.14 (1.00, 1.30)	–	–	1.13 (0.99, 1.30)
	Acquired Immune Deficiency	–	0.38 (0.14, 1.00)	–	–

AK = Above Knee Amputation, BK = Below Knee Amputation, CCI = Charlson's Comorbidity Index, CI = Confidence Interval, FT = Foot or Toe Amputation, HR = Hazard Ratio, KT = Kidney Transplantation, LEA = Lower Extremity Amputation

Supplementary Figure S2. Event free survival curve of the entire group and subgroups

1. **Entire and subgroup survival curve of lower extremity amputation**

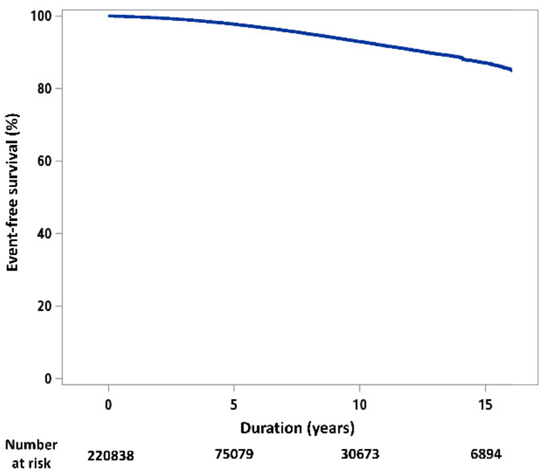

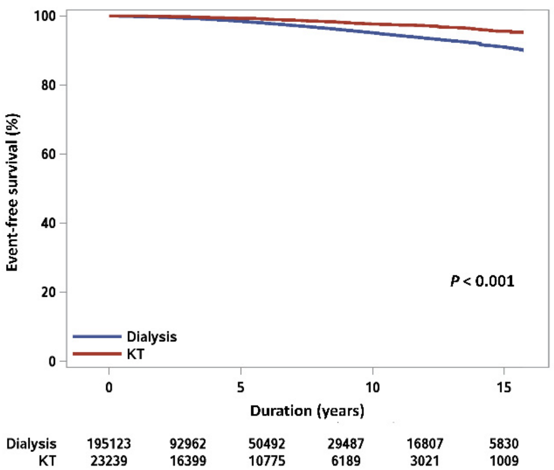

2. Entire and subgroup survival curve of above knee amputation

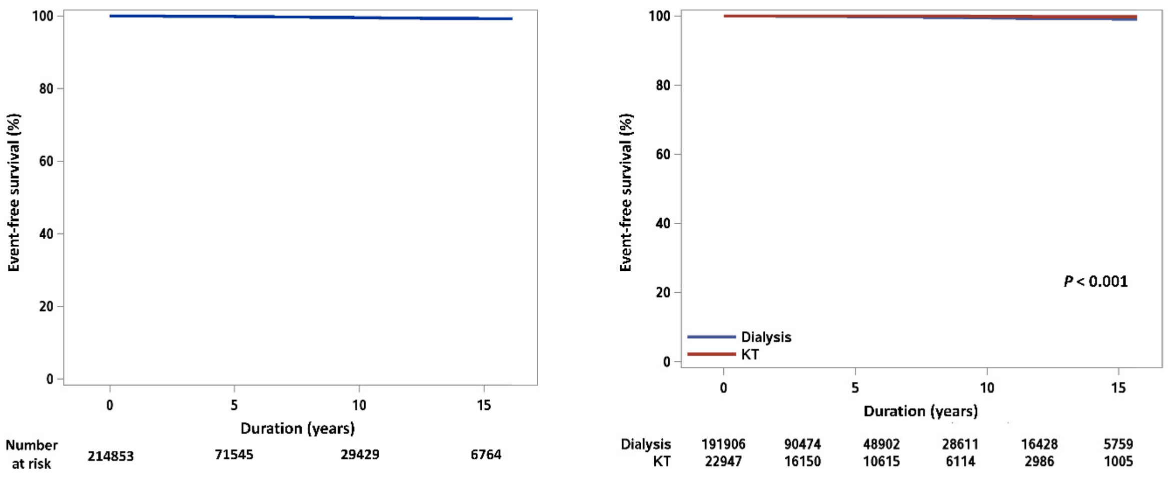

3. Entire and subgroup survival curve of blow knee amputation

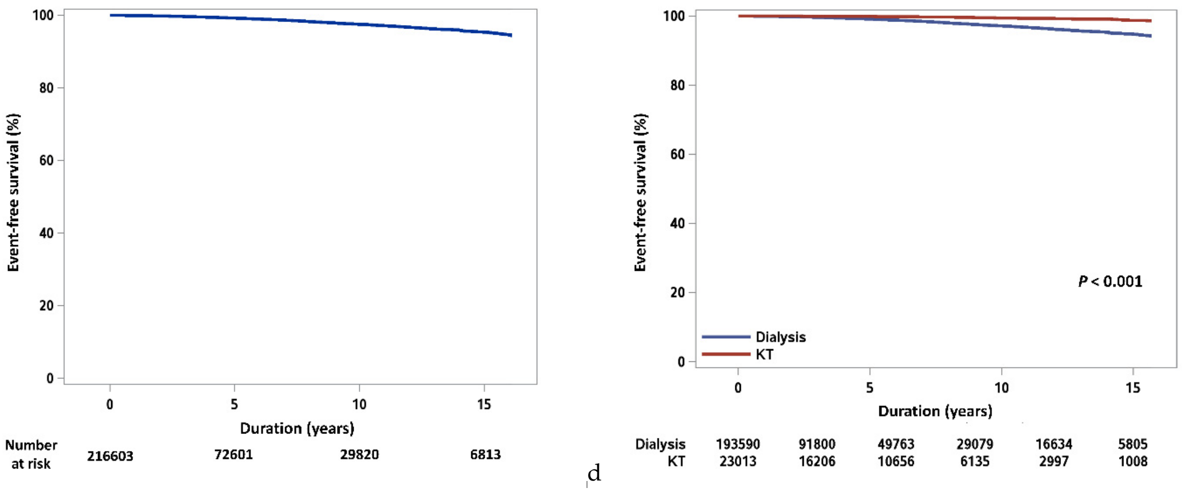

4. Entire and subgroup survival curve of foot and toe amputation

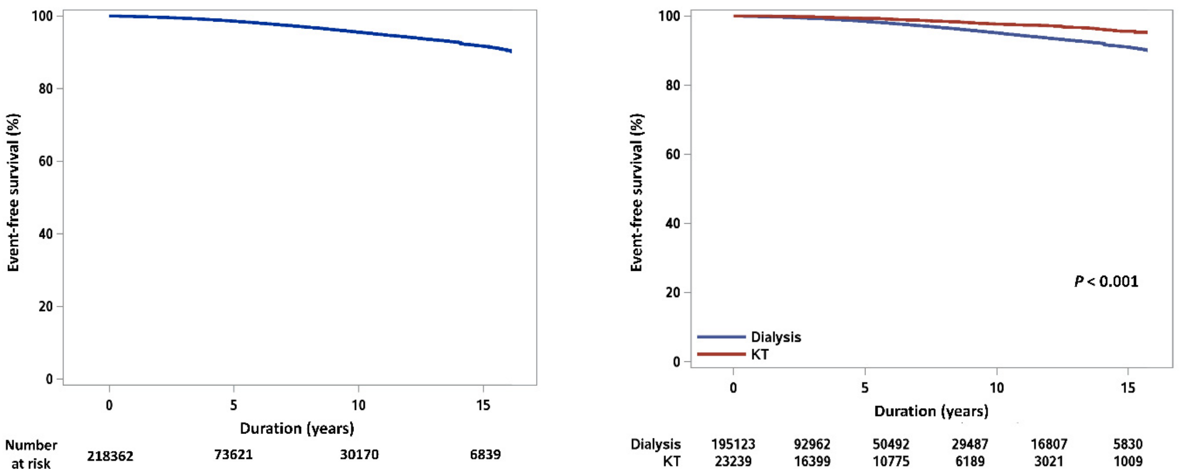

Event-free survival (%)
100
80
60
40
20
0
0
5
10
15
Duration (years)
Number at risk
218362
73621
30170
6839
Event-free survival (%)
P < 0.001
Dialysis
KT
Duration (years)
Dialysis 195123 92962 50492 29487 16807 5830
KT 23239 16399 10775 6189 3021 1009
